# Extensive recombination events and horizontal gene transfer shaped the *Legionella pneumophila *genomes

**DOI:** 10.1186/1471-2164-12-536

**Published:** 2011-11-01

**Authors:** Laura Gomez-Valero, Christophe Rusniok, Sophie Jarraud, Benoit Vacherie, Zoé Rouy, Valerie Barbe, Claudine Medigue, Jerome Etienne, Carmen Buchrieser

**Affiliations:** 1 Institut Pasteur, Biologie des Bactéries Intracellulaires, 75724, Paris, France; 2 CNRS URA 2171, 75724, Paris, France; 3 Université de Lyon, Lyon, France, Centre National de Référence des Legionella, Lyon, France; 4 INSERM, U851, 69007 Lyon, France; 5 Hospices Civils de Lyon, Lyon, France; 6 CEA/DSV/FAR/IG/Genoscope Laboratoire de Génomique Comparative, Evry Cedex, France; 7 CNRS UMR8030 Laboratoire d'Analyses Bioinformatiques en Métabolisme et Génomique, Evry, France

## Abstract

**Background:**

*Legionella pneumophila *is an intracellular pathogen of environmental protozoa. When humans inhale contaminated aerosols this bacterium may cause a severe pneumonia called Legionnaires' disease. Despite the abundance of dozens of *Legionella *species in aquatic reservoirs, the vast majority of human disease is caused by a single serogroup (Sg) of a single species, namely *L. pneumophila *Sg1. To get further insights into genome dynamics and evolution of Sg1 strains, we sequenced strains Lorraine and HL 0604 1035 (Sg1) and compared them to the available sequences of Sg1 strains Paris, Lens, Corby and Philadelphia, resulting in a comprehensive multigenome analysis.

**Results:**

We show that *L. pneumophila *Sg1 has a highly conserved and syntenic core genome that comprises the many eukaryotic like proteins and a conserved repertoire of over 200 Dot/Icm type IV secreted substrates. However, recombination events and horizontal gene transfer are frequent. In particular the analyses of the distribution of nucleotide polymorphisms suggests that large chromosomal fragments of over 200 kbs are exchanged between *L. pneumophila *strains and contribute to the genome dynamics in the natural population. The many secretion systems present might be implicated in exchange of these fragments by conjugal transfer. Plasmids also play a role in genome diversification and are exchanged among strains and circulate between different *Legionella *species.

**Conclusion:**

Horizontal gene transfer among bacteria and from eukaryotes to *L. pneumophila *as well as recombination between strains allows different clones to evolve into predominant disease clones and others to replace them subsequently within relatively short periods of time.

## Background

*Legionella pneumophila *is the etiologic agent of Legionnaires' disease, an atypical pneumonia, which is often fatal if not treated promptly. However, it is principally an environmental bacterium that inhabits fresh water reservoirs worldwide where it parasitizes within free-living protozoa but also survives in biofilms [1-3]. Since *L. pneumophila *does not spread from person-to-person, humans have been inconsequential for the evolution of this pathogen. Instead, the virulence strategies of *L. pneumophila *have been shaped by selective pressures in aquatic ecosystems. Indeed, the co-evolution of *L. pneumophila *with fresh-water amoebae is reflected in its genome sequence. The analysis of two *L. pneumophila *genomes identified the presence of an unexpected high number and variety of eukaryotic-like proteins and proteins containing motifs mainly found in eukaryotes [4]. These proteins were predicted to interfere in different steps of the infectious cycle by mimicking functions of eukaryotic proteins [4]. For several of these eukaryotic like proteins it has been shown recently that they are secreted effectors that help *L. pneumophila *to subvert host functions to allow intracellular replication [5,6]. The possibility that *L. pneumophila *has acquired at least some of these genes through horizontal gene transfer from eukaryotes has been suggested by two studies [7,8].

Plasticity is another specific feature of the *L. pneumophila *genomes as integrative plasmids, putative conjugation elements and genomic islands were identified. In addition to DNA interchange between different bacterial genera and even domains of life, horizontal gene transfer within the genus *Legionella *and within the species *L. pneumophila *has been reported. For example a 65-kb pathogenicity island described first in *L. pneumophila *strain Philadelphia [9] is present in several *L. pneumophila *strains and also in other *Legionella *species like *L. anisa *[10]. Another example is the particular lipopolysaccharide cluster of serogroup 1 strains that has been detected in *L. pneumophila *strains of different lineages and genetic backgrounds [10]. *L. pneumophila *has all necessary features for incorporating foreign DNA, as these bacteria are naturally competent and possess an intact recombination machinery [11,12]. These findings suggest that the *L. pneumophila *genomes are very dynamic and one would expect that horizontal gene transfer and recombination events play an important role in their evolution.

However, different analyses like early studies applying multilocus enzyme electrophoreses (MEE) supported a clonal population structure of *L. pneumophila *[13]. Two recent reports using genetic profiling based on six or three genetic loci, respectively concluded also that *L. pneumophila *shows a clonal populations structure [14,15] although the presence of few recombination events was not ruled out. Later the analysis of the *dotA*, *mip *and *rpoB *genes in different isolates suggested for the first time that recombination may play some role in *L. pneumophila *evolution [16-18] and a more in depth analysis using over 20 loci suggested that recombination events might be more frequent than was previously thought [19]. However, comparisons of these studies are difficult due to different sampling and different analysis methods used. Furthermore there may be a bias associated with some of the genes selected in these studies like intergenic spacer regions or genes under positive selection that may lead to artefactual effects in detecting recombination. To solve these problem efforts have been undertaken recently to homogenize the results obtained for different species to allow comparisons [20]. These authors report for *L. pneumophila *a low recombination rate like for the obligate pathogens *Bordetella pertussis *or *Bartonella henselae*. In contrast Coscolla and colleagues suggest a more important role for recombination at the intergenic level [21].

These different results and the fact that a globally distributed *L. pneumophila *clone implicated in Legionnaires' disease has been described [10] may suggest that the role of recombination is not relevant. However, the description of clonal complexes is not incompatible with high recombination rates. Transient clones may appear within a recombining population [22], in particular if clones with high disease prevalence appear, as this seems to be the case for some *L. pneumophila *strains. These clones are often vastly over-sampled due to their clinical importance and show strong clonality. Thus, this may be correct for this subgroup, but it may not be representative for the population. Indeed when analyzing over 200 clinical and environmental *L. pneumophila *strains, significantly less diversity was found among the clinical isolates [23].

In this study we investigated the genome dynamics and evolution of the species *L. pneumophila *by analyzing horizontal gene transfer, mobile genetic elements and recombination on a genome-wide level. We undertook this analysis based on six complete genome sequences four of which are the previously published reference genomes of *L. pneumophila *Paris, Lens [4], Corby [24] and Philadelphia [25] and two that were sequenced in this study. The newly sequenced strains were selected according to epidemiological features that might be reflected in their genomes and should thus allow to study genome dynamics with respect to virulence. Strain Lorraine is rarely isolated from the environment but its prevalence in human disease is increasing considerably in the last years [26]. In contrast, *L. pneumophila *strain HL 0604 1035 has been frequently isolated from a hospital water system since over 10 years but has never caused disease. Analysis of these six strains identified a highly conserved and syntenic core genome and a diverse accessory genome. Furthermore, it showed that recombination events and horizontal gene transfer are frequent in *L. pneumophila*. Horizontal gene transfer from eukaryotes as well as recombination between strains were identified suggesting that *L. pneumophila *genomes are highly dynamic, a feature allowing different clones to evolve into predominant disease clones and others to replace them subsequently within relatively short periods of time.

## Results and discussion

### The *L. pneumophila *core genome comprises over 2400 conserved genes that are highly syntenic

**Figure 1:**
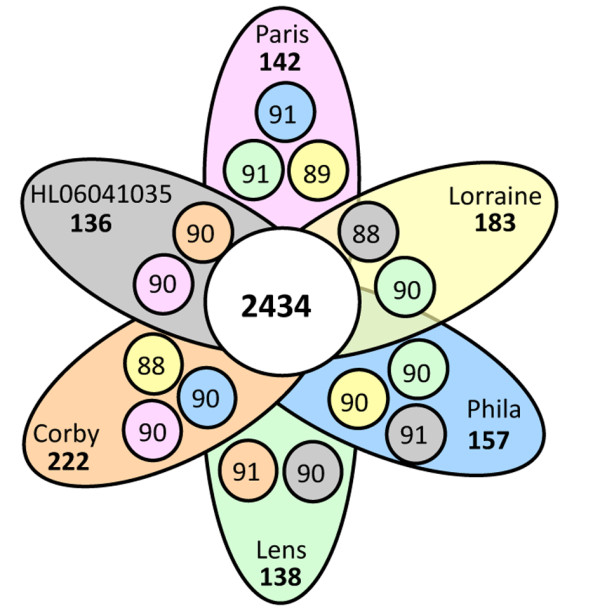
**Shared and specific gene content of 6 *L. pneumophila *genomes**. Each petal represents a genome with an associated color. The number in the center of the diagram represents the orthologous genes shared by all the genomes. The number inside of each individual petal corresponds to the specific genes of each genome with non-orthologous genes in any of the other genomes. The small circles inside of each petal represent the percentage of shared genes (total number divided by the number of genes in the smallest genome) between the genome of this petal and the genome represented by the color of the small circle. Yellow circle inside orange petal means that there are 88% of genes shared among Corby and Lorraine.

To get comprehensive insight into the genetic basis, evolution and genome dynamics of *L. pneumophila *Sg1, the strains responsible for over 90% of disease worldwide, we analyzed six completely sequenced genomes. The strains selected are all of Sg1, have endemic and/or epidemic character (*e.g*. Paris, Lorraine or Philadelphia) were isolated in different countries (France, England, Spain, US) and in different years. Two strains were newly sequenced for this study (Lorraine and HL 0604 1035), the other four *L. pneumophila *genomes (Paris, Lens, Philadelphia, Corby) have been published previously [4,24,25]. The genomes of *L. pneumophila *Lorraine and HL 0604 1035 consist each of a single circular chromosome of 3.4 Mb. Strain Lorraine also contains a plasmid. As shown in Table 1, the main features of the six *L. pneumophila *genomes analyzed (*e.g*. genome size, GC content and coding density), are highly conserved. The core genome of the six *L. pneumophila *genomes comprises 2434 genes, which represents about 80% of the predicted genes in each genome. Furthermore, the gene order is highly conserved as the 260 kb inversion in strain Lens with respect to the other strains is the only exception. When comparing the strains two by two, in average 90% of the genes are present in both strains (Figure 1). However, when determining the non-orthologous genes specific of each genome and not present in the remaining 5 strains, each strain contains between 136 (strain HL 0604 1035) and 222 (strain Corby) strain specific genes mainly encoded on mobile genetic elements. Taken together, the *L. pneumophila *genomes have a highly conserved and syntenic backbone and a highly dynamic accessory genome of about 300 genes each mainly formed by mobile genetic elements, genomic islands and genes of unknown function. The complete annotation of these six genomes is available in a new data base resource that we have set up, LegionellaScope https://www.genoscope.cns.fr/agc/microscope/about/collabprojects.php?P_id = 27 and at the Institut Pasteur, LegioList http://genolist.pasteur.fr/LegioList/.

**Table 1:** General features of the 6 *L. pneumophila *strains analyzed

*L. pneumophila *strains	Philadelphia	Paris	Lens	Corby	HL06041035	Lorraine
**Chromosome size (bp)**	3397754	3503610	3345687	3576469	3492535	3467254
**G+C content (%)**	38.27	38.37	38.42	38.48	38.35	38.36
**N° of genes**	3031	3123	2980	3237	3132	3117
**N° of protein coding genes**	2999	3078	2921	3193	3079	3080
**Pseudogenes**	55	71	84	59	73	48
**tRNA**	43	43	43	44	43	44
**16S/23S/5S**	3/3/3	3/3/3	3/3/3	3/3/3	3/3/3	3/3/3
**Average length CDS (nts)**	1082.47	1000.85	1008.76	984.35	995.47	988.54
**Average length ig (nts)**	147.72	154	152.36	149.24	155.12	155.28
**Coding density (%)**	88.22	86.93	87.07	87.25	86.94	87.26
**Plasmids**	0	1	1	0	0	1

bp, base pairs; nts, nucleotides; CDS, coding sequence; ig, intergenic region

### The species *L. pneumophila *has a highly conserved core genome

#### a) Most eukaryotic like proteins are conserved in all *L. pneumophila *genomes

The presence of proteins with high similarity to eukaryotic proteins or proteins with domains preferentially or only present in eukaryotic genomes are a particular feature of *L. pneumophila *[4]. However, the criteria for identifying these proteins were never clearly defined. To analyze their evolution and possible origin in depth we have thus developed an automatic and systematic method to identify eukaryotic like proteins according to defined criteria. Previously we had identified eukaryotic like proteins in *L. pneumophila *as proteins with the highest similarity score to eukaryotic proteins according to BLAST results, or by identifying eukaryotic domains [4]. However, due to constantly growing databases BLAST results are changing. Furthermore, recent analyses of amoeba-associated bacteria, in particular symbionts of amoeba have shown that they also contain eukaryotic like proteins, suggesting multiple origins of these proteins in prokaryotes [27]. To get a more complete picture of eukaryotic like proteins of *L. pneumophila *and also to include those proteins that might have been transferred independently to different amoeba associated bacteria we defined a eukaryotic like protein as i) a protein having a better normalized blast score against eukaryotic sequences than against prokaryotic ones and ii) a protein that did not show BLAST results against neither *Legionella *spp. nor other bacterial species for which resistance to amoeba infection has been demonstrated (see material and methods). Applying these criteria we identified 46 proteins with putative eukaryotic origin, of which 17 are described here for the first time (Table 2). Given the fact that these proteins were probably acquired by HGT one would expect high diversity in the repertoire. However, our analyses revealed a considerable conservation as more than 50% (26) are conserved in all six *L. pneumophila *strains, indicating an ancient transfer. Furthermore, they show 89-99% nucleotide identity, probably due to high selection pressure for their maintenance. Thus most of these proteins belong to the core genome, indicating that their acquisition has taken place before the speciation of *L. pneumophila*. These 26 proteins might have allowed a common *Legionella *ancestor to colonize an intracellular niche or to adapt better to the intercellular environment of a specific protozoan species leading to the evolution of the species *L. pneumophila*. Interestingly, 19 of these 26 proteins are also conserved in *L. longbeachae*, which might thus be those indispensible for intracellular replication of *Legionellae *(Table 2) [28].

**Table 2:** Orthologous eukaryotic like proteins present in the 6 *L. pneumophila *strains and in *L. longbeachae*

	Name	*L. pneumophila *strains												*L. lo*
**Product**		**Paris**		**Lens**		**Corby**		**Lorraine**		**HL06041035**		**Philadelphia**		

Glucoamylase (Glucan 1,4-alpha-glucosidase)		*lpp0489*	99.31	*lpl0465*	98.93	*lpc2921*	99.38	*lpo0482*	99.45	*lpv0523*	99.14	lpg0422*	95.92	*llo2801*
Putative inosine-uridine nucleoside N-ribohydrolase §		*lpp0208*	98.81	*lpl0206*	98.64	*lpc0223*	98.94							
SidE protein		*lpp0304*	98.46	*lpl0288*	95.25	*lpc1602*	98.35	*lpo0273*	97.95	*lpv0315*	98.28	lpg0234*	94.63	
Putative methyltransferase		*lpp0358*	98.06	*lpl0334*	89.28	*lpc0359*	97.93	*lpo0334*	97.29	*lpv0375*	97.80	*lpg0282*	89.28	*llo2356*
Conserved exported protein of unknown function		*lpp0379*	99.63	*lpl0354*	98.53	*lpc0380*	99.45	*lpo0358*	99.45		99.82	*lpg0301*	99.26	
Phosphatidylcholine-hydrolyzing phospholipase C §		*lpp0565*	99.37	*lpl0541*	98.66	*lpc2843*	99.34	*lpo0571*	99.21	*lpv0603*	99.29	*lpg0502*	97.87	*llo1329*
Phytanoyl-CoA dioxygenase domain-containing protein 1		*lpp0578*	99.25	*lpl0554*	98.60	*lpc2829*	99.46	*lpo0586*	98.60	*lpv0619*	99.25	lpg0515*	99.35	*llo3224*
Leucine-rich repeat protein		*lpp1007*	97.53			*lpc2344*	97.87	*lpo1029*	93.94	*lpv1082*	97.87	lpg0945*	97.54	
ecto-ATP diphosphohydrolase II	*map*	*lpp1033*	98.95	*lpl1000*	98.78	*lpc2316*	98.78	*lpo1060*	98.69	*lpv1110*	98.86	*lpg0971*	98.43	*llo1247*
Major acid phosphatase Map §		*lpp1120*	99.06	*lpl1124*	97.92	*lpc0577*	98.12	*lpo1121*	97.93	*lpv1267*	99.06	*lpg1119*	98.59	*llo1016*
Pyruvate decarboxylase		*lpp1157*	99.70	*lpl1162*	98.87	*lpc0618*	99.70	*lpo1168*	98.69	*lpv1308*	99.70	*lpg1155*	98.51	
SAM-dependent methyltransferase §		*lpp1192*	98.38	*lpl1198*	98.97	*lpc0657*	99.15	*lpo1205*	99.06	*lpv1346*	97.61	*lpg1190*	99.15	*llo1296*
Putative 2OG-Fe(II) oxygenase superfamily protein §		*lpp1405*	100					*lpo1449*	96.84	*lpv1569*	100,00	*lpg1450*	93.74	
Phospholipase C §		*lpp1411*	100	*lpl1573*	93.12	*lpc0870*	100,00	*lpo1455*	97.61	*lpv1576*	100,00	*lpg1455*	97.93	*llo1329*
Putative mitogen-activated protein kinase	*thi*	*lpp1439*	99.12	*lpl1545*	98.11	*lpc0898*	97.61	*lpo1483*	98.93	*lpv1609*	99.12	lpg1483*	97.67	*llo1682*
Thiamine biosynthesis protein NMT-1		*lpp1522*	99.04	*lpl1461*	97.98	*lpc0988*	99.04	*lpo1583*	97.88	*lpv1700*	99.04	*lpg1565*	97.47	*llo0920*
Leucine-rich repeat-containing protein	*purC*	*lpp1567*	97.68			*lpc1028*	98.44			*lpv1852*	98.91	lpg1602*	97.98	
Phosphoribosylamidoimidazole-succinocarboxamide synthase	*mvaB*	*lpp1647*	100	*lpl1640*	97.76	*lpc1106*	99.08	*lpo1715*	98.98	*lpv1936*	98.16	*lpg1675*	97.58	*llo3277*
Hydroxymethylglutaryl-CoA lyase (HMG-CoA lyase) §		*lpp1793*	99.34	*lpl1794*	97.13	*lpc1274*	98.01	*lpo1891*	99.01	*lpv2102*	98.68	*lpg1830*	99.12	*llo0113*
Putative apyrase		*lpp1880*	99.47	*lpl1869*	98.77	*lpc1359*	99.74	*lpo1975*	98.86	*lpv2179*	99.56	*lpg1905*	95.34	*llo1247*
Conserved protein of unknown function		*lpp1905*												
Leucine-rich repeat-containing protein		*lpp1940*	94.44					*lpo2043*	93.7	*lpv2255*	93.88	lpg1958*	92.56	
ZIP metal transporter family protein §		*lpp2018*	99.60	*lpl2013*	99.07	*lpc1521*	99.47	*lpo2138*	99.34	*lpv2339*	99.47	*lpg2035*	99.07	*llo2518*
Ankyrin repeat protein		*lpp2058*	99.2	*lpl2048*	90.42	*lpc1566*	98.80	*lpo2181*	98.05					
Conserved protein of unknown function		*lpp2061*	99.6	*lpl2051*	95.95	*lpc1569*	96.80	*lpo2185*	95.38					
Sphingosine-1-phosphate lyase I		*lpp2128*	98.84	*lpl2102*	98.29	*lpc1635*	99.06	*lpo2245*	98.62	*lpv2428*	98.02	lpg2176*	94.02	
Conserved protein of unknown function		*lpp2134*	100	*lpl2109*	98.55	*lpc1642*	100	*lpo2253*	99.60	*lpv2436*	100	*lpg2182*	96.27	
Conserved protein of unknown function		*lpp2419*	99.84	*lpl2298*	99.37	*lpc2129*	100							
Leucine rich repeat protein		*lpp2459*	98.98	*lpl2316*	86.85	*lpc2085*	90.48	*lpo2572*	99.43	*lpv2704*	99.32	lpg2392*	97.28	
Putative unspecific monooxygenase		*lpp2468*	99.47	*lpl2326*	99.01	*lpc2075*	98.88	*lpo2586*	98.15					
Protein kinase-like		*lpp2626*	94.88	*lpl2481*	98.85	*lpc1906*	95.31	*lpo2765*	98.70	*lpv2900*	99.13	lpg2556*	99.13	*llo2218*
Putative methyltransferase		*lpp2747*	99.25	*lpl2620*	99	*lpc0443*	99.37	*lpo2974*	97.49	*lpv3039*	99.62	*lpg2693*	99.37	*llo2356*
Phytanoyl-CoA dioxygenase, PhyH		*lpp2748*	99.76	*lpl2621*	98.91	*lpc0442*	99.15	*lpo2975*	98.67	*lpv3040*	99.76	lpg2694*	95.44	
Sugar kinase §	*hemG*	*lpp2874*	99.38			*lpc3108*	98.89	*lpo3114*	98.15	*lpv3175*	99.14	*lpg2821*	98.52	
Protoporphyrinogen oxidase §	*cysK*	*lpp2909*	98.14	*lpl2763*	96.65	*lpc3136*	98.90	*lpo3153*	98.69	*lpv3207*	98.83	*lpg2851*	96.02	*llo0133*
Cysteine synthase A, O-acetylserine sulfhydrolase A subunit		*lpp3022*	99.26	*lpl2880*	98.95	*lpc3266*	95.99	*lpo3279*	99.16	*lpv3334*	99.26	*lpg2951*	98.52	*llo0076*
Putative methyltransferases §		*lpp3025*	98.50	*lpl2883*	97.06	*lpc3269*	99.30	*lpo3282*	97.62	*lpv3338*	97.54	*lpg2954*	97.76	*llo0074*
Flavanone 3-dioxygenase §								*lpo1380*						
Protein of unknown function §								*lpo1577*						
Conserved protein of unknown function with SNARE domain §						*lpc2110*	98.97	*lpo2553*	97.25	*lpv2681*	98.97			
(S)-2-hydroxy-acid oxidase §								*lpo2960*						
Protein of unknown function §								*lpo3145*	100,00	*lpv3199*	94.82			
Putative Pyridine nucleotide-disulphide oxidoreductase				*lpl2845*	95.59	*lpc3225*	97.70	*lpo3239*	98.47	*lpv3288*	97.80	*lpg2917*	98.28	
Regulator of chromosome condensation, rcc										*lpv2481*	79.24	lpg2224*	99.83	
Putative metallophosphoesterase §										*lpv2663*				
Serine carboxypeptidase										*lpv3278*	97.64	*lpg2911*	100	

* Substrates of the Dot/Icm secretion system; § eukaryotic like proteins newly identified in this study; numbers, % nucleotide identity to strain Philadelphia; L.lo, *Legionella longbeachae*

#### b) Eukaryotic protein motifs are highly conserved among the *L. pneumophila *genomes

A second class of eukaryotic proteins of *L. pneumophila *is carrying domains predominantly present in eukaryotic proteins. To systematically identify these proteins we used the Interpro database comprising 10 different domain search programs [29]. This allowed to identify the *L. pneumophila *proteins carrying eukaryotic domains in the newly sequenced strains Lorraine and HL 0604 1035 as well as to identify previously not reported motifs. Similarly to the above described eukaryotic like proteins over half of the eukaryotic domain coding proteins are conserved in all six genomes and over 80% are conserved when two genomes are compared *(e.g*. 33 of the 39 proteins containing an eukaryotic motif in strain Lens are present also in strain Paris). Moreover half of them share very high nucleotide identity of in average 98%-100% (Table 3) again suggesting high selection pressure to maintain them.

**Table 3:** Orthologous proteins with eukaryotic motifs present in the 6 *L. pneumophila *strains and in *L. longbeachae *

Motif	*L. pneumophila *strains												*L. lo*
	**Paris**		**Lens**		**Philadelphia**		**Lorraine**		**HL06041035**		**Corby**		

ANK	*lpp0037*	96.30	*lpl0038*	97.40	lpg0038*	97.04	*lpo0042*	97.89	*lpv0043*	93.66	*lpc0039*	97.10	
ANK	*lpp0126*	98.94	*lpl0111*	98.48	*lpg0112*	94.83	*lpo0119*	98.79	*lpv0127*	93.03	*lpc0131*	92.16	*llo1394*
ANK	*lpp0202*												
ANK	*lpp0356*												
ANK	*lpp0469*	98.94	*lpl0445*	96.35	lpg0403*	95.53	*lpo0463*	97.64	*lpv0501*	98.48	*lpc2941*	98.67	
ANK	*lpp0503*	98.37	*lpl0479*	93.86	lpg0436*	93.31	*lpo0501*	98.12	*lpv0537*	98.37	*lpc2906*	98.37	
ANK	*lpp0547*	99.50	*lpl0523*	96.31	lpg0483*	96.82	*lpo0551*	99.83	*lpv0585*	98.16	*lpc2861*	99.16	*llo2705*
ANK	*lpp0750*	100.00	*lpl0732*	97.65	lpg0695*	100.00	*lpo0775*	99.84	*lpv0817*	100.00	*lpc2599*	98.44	
ANK	*lpp1100*												
ANK + SET	*lpp1683*	97.68	*lpl1682*	96.32	lpg1718*	98.41	*lpo1757*	97.86	*lpv1985*	96.91	*lpc1152*	97.25	
ANK	*lpp1905*												
ANK	*lpp2058*	99.20	*lpl2048*	90.42			*lpo2181*	98.05			*lpc1566*	98.80	
ANK	*lpp2061*	99.60	*lpl2051*	95.95			*lpo2185*	95.38			*lpc1569*	96.80	
ANK	*lpp2065*	99.93	*lpl2055*	98.56			*lpo2189*	98.62			*lpc1573*	98.03	
ANK + Fbox	*lpp2082*	97.40	*lpl2072*	98.26	lpg2144*	98.84	*lpo2207*	99.03	*lpv2392*	93.99	*lpc1593*	99.22	
ANK	*lpp2166*	99.25	*lpl2140*	99.12	lpg2215*	99.06	*lpo2285*	97.74	*lpv2469*	99.18	*lpc1680*	98.93	
ANK	*lpp2248*	99.50	*lpl2219*	99.14	lpg2300*	99.14	*lpo2371*	99.43	*lpv2567*	98.93	*lpc1765*	99.21	*llo0584*
ANK	*lpp2270*	99.64	*lpl2242*	97.97	lpg2322*	98.34	*lpo2399*	98.08	*lpv2591*	99.53	*lpc1789*	99.53	*llo0570*
ANK	*lpp2517*	99.60	*lpl2370*	97.94	lpg2452*	98.19	*lpo2642*	98.95	*lpv2776*	99.46	*lpc2026*	99.46	
ANK	*lpp2522*	98.76	*lpl2375*	96.90	lpg2456*	95.75	*lpo2647*	98.49	*lpv2781*	98.76	*lpc2020*	91.27	*llo0365*
ANK	*plpp0098*	96.00					*lpop0045*	96.00					
ANK			*lpl1681*	100.00							*lpc1151*	97.98	
ANK			*lpl2058*	86.17			*lpo2193*	95.37	*lpv2375*	94.96			
ANK			*lpl2339*	98.64	lpg2416*	91.21	*lpo2601*	99.00	*lpv2736*	99.28	*lpc2057*	98.98	
ANK					lpg0402*	100.00			*lpv0500*	96.01			
ANK									*lpv2258*				
ANK			*lpl1681*	100.00							*lpc1151*	97.98	
ANK			*lpl2344*	100.00			*lpo2607*	97.93					

F-Box	*lpp0233*	98.58	*lpl0234*	93.97	lpg0171*	96.81	*lpo0202*	97.87	*lpv0254*	98.94			
F-Box	*lpp2486*												
F-Box					lpg2224*	99.83			*lpv2482*	79.24			
F-Box									*lpv2481*				

RAS GEF	*lpp0350*	94.53	*lpl0328*	96.32	lpg0276*	97.33	*lpo0327*	97.64	*lpv0368*	97.64	*lpc0353*		*llo0327*
RAS GEF§									*lpv2258*				

Sec7	*lpp1932*	98.41	*lpl1919*	97.40	lpg1950*	92.16	*lpo2033*	98.32	*lpv2243*	98.58	*lpc1423*	97.57	*llo1397*

Sel1											*lpc0165*		
Sel1			*lpl1059*	100.00	*lpg1062*	99.61			*lpv1209*	100.00	*lpc2212*	99.61	
Sel-1§	*lpp0957*	98.93	*lpl0927*	98.67	*lpg0896*	98.93	*lpo0978*	98.67	*lpv1030*	98.67	*lpc2397*	99.47	*llo0844*
Sel-1	*lpp1174*	99.39	*lpl1180*	98.30	*lpg1172*	98.32	*lpo1187*	99.11	*lpv1327*	99.39	*lpc0638*	99.05	
Sel-1	*lpp1310*	97.87	*lpl1307*	98.40	*lpg1356*	98.67	*lpo1345*	99.02	*lpv1469*	97.87	*lpc0770*	98.76	*llo1443*
Sel-1	*lpp2174*	99.64	*lpl2147*	98.48	lpg2222*	99.56	*lpo2292*	99.47	*lpv2477*	99.47	*lpc1689*	96.27	
Sel-1	*lpp2692*	99.25	*lpl2564*	98.61	*lpg2639*	98.39	*lpo2917*	99.28	*lpv2979*	99.39	*lpc0501*	98.75	*llo2649*
Sel-1							*lpo3233*						

Spectrin	*lpp1848§*	99.18	*lpl1845*	98.77	lpg1884*	99.01	*lpo1944§*	98.93	*lpv2158§*	99.18	*lpc1331*	99.18	
Spectrin	*lpp2246*	99.29	*lpl2217*	98.75	lpg2298*	99.29	*lpo2369*	99.29	*lpv2565*	98.27	*lpc1763*	98.75	*llo1707*
Spectrin	*lpp1930*	95.11			lpg1947*	96.65	*lpo2029*	97.72					
Spectrin	*lpp1309*	100.00			lpg1355*	90.59			*lpv1468*	100.00			
Spectrin ^§^	*lpp1002*	98.01	*lpl0971*	91.62	lpg0940*	97.92	*lpo1024*	98.05	*lpv1077*	97.87	*lpc2349*	97.15	
Spectrin §	*lpp0471*	97.79	*lpl0447*	97.45	lpg0405*	98.30	*lpo0465§*	98.28	*lpv0504*	98.28	*lpc2939*	97.70	*llo2845*
Spectrin §	*lpp1843*	95.45	*lpl1840*	97.57					*lpv2151*	100.00	*lpc1323§*	99.60	
Spectrin §	*lpp1173*	98.56	*lpl1179§*	98.80	lpg1171*§	98.56	*lpo1186*	99.28	*lpv1326*	98.56	*lpc0637*	97.84	*llo3114*
STPK	*lpp0267*	96.95	*lpl0262*	98.72	*lpg0208*	93.26	*lpo0242*	98.92	*lpv0288*	95.13	*lpc0283*	97.26	
STPK	*lpp1439*	99.12	*lpl1545*	98.11	lpg1483*	97.67	*lpo1483*	98.93	*lpv1609*	99.12	*lpc0898*	97.61	*llo1682*
STPK	*lpp2626*	94.88	*lpl2481*	98.85	lpg2556*	99.13	*lpo2765*	98.70	*lpv2900*	99.13	*lpc1906*	95.31	*llo2218*
U-box	*lpp2887*	99.72			lpg2830*	97.15	*lpo3124*	99.58	*lpv3185*	98.75			

*Substrates of the Dot/Icm secretion system according to previous publications; ^¶ ^orthologs proteins where the corresponding motif was not present in the other genome; ^§ ^eukaryotic like proteins newly identified in this study; numbers, nucleotide identity with respect to the *L. pneumophila *Philadelphia gene; *L.lo*, *Legionella longbeachae*

Our approach identified also new eukaryotic domains like spectrin repeats. The spectrin repeat forms a three-helix bundle and was reported primarily in the animal kingdom [30]. These repeats act as modules building long, extended molecules that also serve as a docking surface for cytoskeletal and signal transduction proteins. In *L. pneumophila *it is present in up to eight proteins of each strain (Table 3) and all spectrin repeat proteins are predicted to be secreted Dot/Icm substrates [31-33]. Another interesting domain is the RAS GEF domain that is present in two proteins encoded by strain Paris one of which (Lpp0350) is conserved in the six strains analyzed. Ras-GEFs are small GTPases typically present in eukaryotes that are involved in numerous cellular processes like gene expression, cytoskeleton re-organization, microtubule organization and vesicular and nuclear transport [34]. GEFs (GDP-GTP exchange factors) regulate Rabs, GTP-binding proteins with conserved functions in membrane trafficking [35]. Interestingly, according to the Pfam database Ras-GEF domains in bacteria are only present in *Legionella*, *Parachlamydia acanthamoebae *and *Protochlamydia amoebophila*, all of which are amoeba-associated bacteria.

Coiled-coil domains have been identified previously in the *L. pneumophila *genomes as this motif can be found in all kingdoms of life. However extended coiled-coil domains are largely absent from bacterial genomes but are typical for archaea and eukaryotes. We thus searched the *L. pneumophila *genomes and 29 other genomes of bacterial pathogens or bacteria present in the aquatic environment (Table 4) for proteins with five or more coiled coil domains. Interestingly, *Legionella *spp, *Streptococcus pneumoniae *and *Pseudomonas aeruginosa *contain the highest percentage of proteins with extended coiled-coil domains (6-11 domains) compared to the number of predicted proteins encoded in their genome and only *P. aeruginosa *and *L. pneumophila *encode proteins containing more than 10 coiled-coil domains (Table 4). Most of these *Legionella *proteins are predicted substrates of the Dot/Icm secretion system [31-33,36]. This suggests that large coiled-coil domains are specific adaptations to the eukaryotic cell probably implicated in interactions with host proteins.

**Table 4:** Genes coding for proteins with more than 5 coiled coil domains/protein in different bacterial genomes

Organism	Coiled coil domains proteins	Gene	Product	Number of Coiled coil
*B. henselae *Houston-1	0			

*Ch. pneumoniae *J138	0			

*Ch. trachomatis *D UW-3	0			

*C.glutamicum *ATCC 13032	0			

*E. coli *O157:H7	1	*ECH74115_2173*	tail length tape measure protein	5

*H. influenzae *Rd KW20	0			

*H. pylori *26695	1	*HP0527*	cag pathogenicity island protein Y	10

*L. pneumophila *Corby	7	*lpc1130*	substrate of the Dot/Icm system/Icm system	5
		*lpc1131*	substrate of the Dot/Icm system/Icm system	6
		*lpc1452*	substrate of the Dot/Icm system/Icm system	6
		*lpc1611*	hypothetical protein	12
		*lpc1987*	substrate of the Dot/Icm system, effector protein B	9
		*lpc2349*	substrate of the Dot/Icm system, LidA	6
		*lpc3079*	substrate of the Dot/Icm system, effector protein A	5

*L. pneumophila *HL06041035	10	*lpv1077*	substrate of the Dot/Icm system, LidA	6
		*lpv1725*	substrate of the Dot/Icm system	6
		*lpv1966*	substrate of the Dot/Icm system	5
		*lpv1967*	substrate of the Dot/Icm system	6
		*lpv2269*	substrate of the Dot/Icm system	7
		*lpv2408*	conserved protein of unknown function	5
		*lpv2816*	substrate of the Dot/Icm system, effector protein B	10
		*lpv2959*	chromosome segregation SMC protein	9
		*lpv3144*	substrate of the Dot/Icm system, effector protein A	5
		*lpv3184*	substrate of the Dot/Icm system, SidH	9

*L. pneumophila *Lens	7	*lpl1437*	substrate of the Dot/Icm system	6
		*lpl1660*	substrate of the Dot/Icm system	7
		*lpl1661*	substrate of the Dot/Icm system	6
		*lpl1941*	substrate of the Dot/Icm system	5
		*lpl2084*	substrate of the Dot/Icm system	5
		*lpl2411*	substrate of the Dot/Icm system, effector protein B	9
		*lpl2708*	substrate of the Dot/Icm system, effector protein A	5

*L. pneumophila *Lorraine	10	*lpo1024*	substrate of the Dot/Icm system, LidA	6
		*lpo1608*	substrate of the Dot/Icm system	6
		*lpo1735*	substrate of the Dot/Icm system	7
		*lpo1736*	substrate of the Dot/Icm system	5
		*lpo2060*	substrate of the Dot/Icm system	6
		*lpo2216*	substrate of the Dot/Icm system, SdeC	5
		*lpo2680*	substrate of the Dot/Icm system, effector protein B	9
		*lpo2896*	chromosome segregation SMC protein	9
		*lpo3083*	substrate of the Dot/Icm system, effector protein A	5
		*lpo3123*	substrate of the Dot/Icm system	9

*L. pneumophila *Paris	6	*lpp1002*	substrate of the Dot/Icm system, LidA	6
		*lpp1546*	substrate of the Dot/Icm system	6
		*lpp1666*	substrate of the Dot/Icm system	7
		*lpp1952*	substrate of the Dot/Icm system	6
		*lpp2555*	substrate of the Dot/Icm system, effector protein B	10
		*lpp2883*	substrate of the Dot/Icm system	6

*L. pneumophila *Philadelphia	8	*lpg1355*	substrate of the Dot/Icm system, SidG protein	5
		*lpg1588*	substrate of the Dot/Icm system	6
		*lpg1701*	substrate of the Dot/Icm system	5
		*lpg1702*	substrate of the Dot/Icm system	6
		*lpg2156*	protein of unknown function	5
		*lpg2490*	substrate of the Dot/Icm system, effector protein B	9
		*lpg2793*	substrate of the Dot/Icm system, effector protein A	5
		*lpg2829*	substrate of the Dot/Icm system	8

*L. monocytogenes *EGD-e	3	*lmo0650*	hypothetical protein	5
		*lmo0955*	hypothetical protein	5
		*lmo1224*	hypothetical protein	5

*M. tuberculosis *F11	1	*TBFG_12936*	chromosome partitioning protein Smc	10

*M. tuberculosis *H37Ra	1	*MRA_2947*	putative chromosome segregation Smc	10

*N. meningitidis *MC58	0			

*P. aeruginosa *LESB58	11	*PLES_08211*	putative tail length tape measure protein	7

		*PLES_12531*	hypothetical protein	7
		*PLES_12541*	hypothetical protein	5
		*PLES_13581*	putative tail length tape measure protein	7
		*PLES_15241*	electron transport complex protein RnfC	8
		*PLES_15871*	hypothetical protein	6
		*PLES_36651*	putative ClpA	_
		*PLES_38011*	putative chromosome segregation protein	11
		*PLES_46621*	putative exonuclease	13
		*PLES_50721*	hypothetical protein	6
		*PLES_55491*	putative outer membrane protein precursor	5

*R. felis *URRWXCal2	2	*RF_0022*	putative surface cell antigen sca1	7
		*RF_0725*	antigenic heat-stable 120 kDa protein	5

*R.prowazekii *Madrid E	0			

*R.a typhi *Wilmington	0			

*S. typhimurium *LT2	5	*STM0395*	exonuclease subunit SbcC	7

		*STM0567*	putative DNA repair ATPase	7
		*STM0994*	chromosome partition protein mukB	10
		*STM1041*	minor tail protein	5
		*STM3199*	hypothetical protein	5

*S. flexner*i 2a 2457T	1	*S0984*	fused chromosome partitioning protein	10

*Synechocystis sp*. PCC 6803	2	*sll1772*	MutS2 protein	5
		*slr1301*	hypothetical protein	6

*S. pneumoniae *D39	4	*SPD_0126*	exported protein of unknown function	6

		*SPD_0710*	putative Septation ring formation regulator EzrA	7
		*SPD_1104*	chromosome partition protein Smc	10
		*SPD_2017*	exported protein of unknown function	6

*W. pipientis *wMel	0			

*X. fastidiosa *9a5c	0			

*Y. pestis *KIM	4	*y0227*	hypothetical protein	6

		*y0976*	ATP-dependent dsDNA exonuclease	12
		*y2765*	chromosome partition protein MukB	10
		*yapB*	autotransporter	6

#### c) High selection pressure acts on the Dot/Icm T4SS and its substrates

Central to the pathogenesis of *L. pneumophila *are the *dot/icm *loci, which together direct assembly of a type IV secretion apparatus [37,38]. Although all *L. pneumophila *strains investigated to date contain the complete *dot/icm *loci, sequence variations among the *dot/icm *genes among different *L. pneumophila *strains have been reported [39]. The *dot/icm *loci of the six strains analyzed here exhibited a very high nucleotide conservation of 98-100% among orthologs except for *dotA*, *icmX *and for *icmC *of strain Corby that is shorter and more divergent (84% nucleotide identity) as compared to *icmC *of strain Paris. These results indicate that strong negative selection acts on these genes (Table 5).

**Table 5:** Percentage of nucleotide identity of orthologous *dot/icm *genes with respect to the *L. pneumophila *Philadelphia sequence

Gene name	Length (nts)	Phila	Paris	Id	Lens	Id	Lorrain	Id	HL06041035	Id	Corby	Id	*L. long*	Id
*icmT*	261	*lpg0441*	*lpp0507*	99.6	*lpl0483*	99.1	*lpo0507*	100	*lpv0541*	96	*lpc2902*	99.2	*llo2795*	75.2
*icmS*	345	*lpg0442*	*lpp0508*	98.5	*lpl0484*	98.8	*lpo0508*	99.1	*lpv0542*	94.4	*lpc2901*	98.3	*llo2794*	76.9
*icmR*	363	*lpg0443*	*lpp0509*	96.9	*lpl0485*	98.3	*lpo0509*	97.8	*lpv0543*	97.5	*lpc2900*	96.9		
*IcmQ*	576	*lpg0444*	*lpp0510*	97	*lpl0486*	99	*lpo0510*	98	*lpv0544*	98	*lpc2899*	98	*llo2792*	70.7
*icmP/dotM*	1131	*lpg0445*	*lpp0511*	98	*lpl0487*	99	*lpo0511*	98	*lpv0545*	98	*lpc2898*	99	*llo2791*	74.5
*icmO/dotL*	2352	*lpg0446*	*lpp0512*	98.4	*lpl0488*	97.7	*lpo0512*	98.1	*lpv0546*	98.3	*lpc2897*	98.3	*llo2790*	77.7
*IcmN/DotK*	570	*lpg0447*	*lpp0513*	99.3	*lpl0489*	98.6	*lpo0513*	98.9	*lpv0547*	99.6	*lpc2896*	99.7	*llo2789*	67.3
*icmM/dotJ*	285	*lpg0448*	*lpp0514*	97.9	*lpl0490*	97.9	*lpo0514*	97.9	*lpv0548*	99.3	*lpc2895*	98.6	*llo2788*	61.7
*icmL/dotI*	639	*lpg0449*	*lpp0515*	99.8	*lpl0491*	99.4	*lpo0515*	99.4	*lpv0549*	99.8	*lpc2894*	99.5	*llo2787*	78.6
*icmK/dotH*	1083	*lpg0450*	*lpp0516*	94.8	*lpl0492*	94.3	*lpo0516*	95.2	*lpv0550*	94.4	*lpc2893*	94.7	*llo2786*	71.2
*icmE/dotG*	3147	*lpg0451*	*lpp0517*	93.7	*lpl0493*	94.0	*lpo0517*	94	*lpv0551*	94	*lpc2892*	94.3	*llo2785*	69.1
*icmG/dotF*	810	*lpg0452*	*lpp0518*	98	*lpl0494*	97	*lpo0518*	98	*lpv0552*	98	*lpc2891*	97	*llo2784*	55.7
*icmC/dotE*	585	*lpg0453*	*lpp0519*	99.6	*lpl0495*	99.1	*lpo0519*	99.7	*lpv0553*	99.3	*lpc2890*	54	*llo2783*	69.1
*icmD/DotP*	399	*lpg0454*	*lpp0520*	97	*lpl0496*	98	*lpo0520*	97	*lpv0554*	98	*lpc2889*	97	*llo2782*	77.3
*icmJ/dotN*	627	*lpg0455*	*lpp0521*	99	*lpl0497*	98	*lpo0521*	99	*lpv0555*	99	*lpc2888*	98	*llo2781*	79.4
*IcmB/DotO*	3030	*lpg0456*	*lpp0522*	98.1	*lpl0498*	98.3	*lpo0522*	98.3	*lpv0556*	98.2	*lpc2887*	97.6	*llo2780*	76.4
*IcmF*	2922	*lpg0458*	*lpp0524*	98.2	*lpl0500*	98.5	*lpo0524*	98.3	*lpv0558*	98.5	*lpc2885*	98.2	*llo3075*	69.5
*IcmH/DotU*	786	*lpg0459*	*lpp0525*	99.4	*lpl0501*	99.5	*lpo0525*	99.7	*lpv0559*	99	*lpc2884*	99	*llo3074*	68.8
*dotD*	492	*lpg2674*	*lpp2728*	98	*lpl2601*	98	*lpo2953*	98	*lpv3018*	98	*lpc0463*	99	*llo0369*	76.5
*dotC*	912	*lpg2675*	*lpp2729*	98.7	*lpl2602*	98.5	*lpo2954*	98.8	*lpv3019*	98.6	*lpc0462*	99.9	*llo0368*	74.8
*dotB*	1134	*lpg2676*	*lpp2730*	99	*lpl2603*	98	*lpo2955*	98	*lpv3020*	98	*lpc0461*	99	*llo0367*	76
*dotA*	3108	*lpg2686*	*lpp2740*	83.3	*lpl2613*	96.8	*lpo2967*	83	*lpv3032*	83.6	*lpc0450*	85.8	*llo0364*	51.4
*icmV*	456	*lpg2687*	*lpp2741*	91	*lpl2614*	91	*lpo2968*	91	*lpv3033*	92	*lpc0449*	92	*llo0363*	64.3
*icmW*	456	*lpg2688*	*lpp2742*	95.1	*lpl2615*	97.6	*lpo2969*	95.1	*lpv3034*	95.4	*lpc0448*	95.1	*llo0362*	79.3
*icmX*	1419	*lpg2689*	*lpp2743*	84.3	*lpl2616*	85.2	*lpo2970*	85.6	*lpv3035*	85.6	*lpc0447*	84.1	*llo0361*	46.9

Id, identity

Since the identification of RalF [40], numerous approaches have been used to identify Dot/Icm translocated substrates. Currently 278 proteins of *L. pneumophila *have been described as being transloctaed by the Dot/Icm T4SS system [7,31,32,41-44]. Analysis of their distribution among the six *L. pneumophila *strains reveals a very high conservation, as 206 of the 278 substrates are present in all six strains. Nearly all of them show a nucleotide similarity of 95-100% and only nine are specific to strain Philadelphia (Additional file 1, Table S1). Furthermore, only 34 of the 278 substrates of strain Philadelphia are missing in strain Paris, 30 in strain Lorraine or 25 in strain HL 0604 1035 (Additional file 1, Table S1). Thus, although high redundancy seems to be present in the repertoire of Dot/Icm effectors, the strong conservation of nearly all of them in all genomes, argues for their mutual importance for the *L. pneumophila *life cycle,

Rare exceptions are RalF and AnkB/Lpp2028. The nucleotide sequence of *ralF *of strain Philadelphia is only 85% similar to the *ralF *genes of the other strains and is 72 nts (24aa) shorter. A similar situation is seen for *lpg2144/ankB *that is 54 nts (18aa) longer in strain Philadelphia and Lens than in strain Paris and Corby. This is surprising, as the C-terminal region of AnkB of strain Philadelphia contains a eukaryotic prenylation C*AAX *motif mediating posttranslational modification of effector proteins, important for intracellular replication of *L. pneumophila*. Lipidation facilitates the localization of this effector protein to host organelles and serves as a docking platform for ubiquitinated proteins [45,46]. Thus in strain Paris and Corby other proteins might take over this function. Taken together, this analysis suggests that over 200 of the Dot/Icm substrates of *L. pneumophila *have been present or have been acquired before the speciation and that such a large repertoire of effectors is indeed necessary for intracellular replication and adaptation to the specific protozoan hosts.

### The species *L. pneumophila *has a highly dynamic accessory genome

#### a) A wide variety of T4ASSs and conjugative elements contribute to genome plasticity

**Figure 2:**
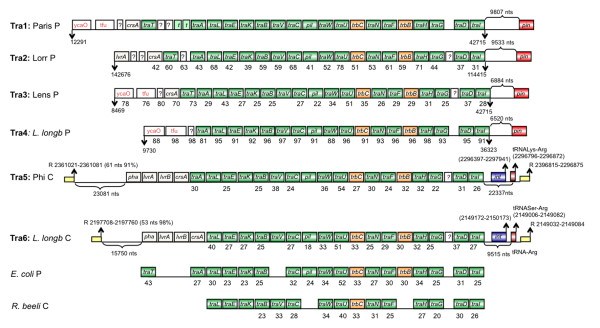
**Schematic representation of F-type IV secretion systems (T4SSA) for conjugal DNA transfer of *L. pneumophila***. In green and orange, *tra *and *trb *genes respectively. L. long, *Legionella longbeachae; *P, Plasmid; C, Chromosome; ycaO, Protein of unknown function with a YcaO like-domain; tfu, Protein of unknown function with a TfuA domain; *pil*, Pilus assembly protein precursor; t, transposase; *E. coli*, *Escherichia coli; R. beeli*, *Rickettsia beeli; pha*, Phage repressor; *int*, integrase*; pin*, site-specific DNA recombinase e14 prophage; R; repeat. Yellow squares represent flanking repeats, with length and percentage of identity between repeats in parenthesis. tRNAs, position in the genome in parenthesis.

**Figure 3:**
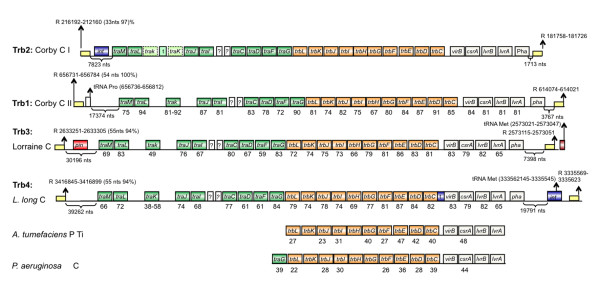
**Schematic representation of P-type IV secretion systems (T4SSA) for conjugal DNA transfer of *L. pneumophila***. In green and orange, *tra *and *trb *genes respectively. L. long, *Legionella longbeachae; *P, Plasmid; C, Chromosome; ?, Protein of unknown function; *A. tumefaciens*, *Agrobacterium tumefaciens; P. aeruginosa, Pseudomonas aeruginosa; *t, transposase; *pha*, Phage repressor; Int*, integrase; *Pseudogenes are in discontinues squares; Yellow squares represent flanking repeats, with length and percentage of identity between repeats in parenthesis. tRNAs, their position in the genome is given in parenthesis.

Based on sequence comparisons, T4SSs are categorized according to their similarity to the *A. tumefaciens *VirB/D4 system into type IVA (type F and P) and type IVB secretion systems [47]. T4ASSs resemble the VirB/D4 system of *A. tumefaciens*, whereas T4BSS proteins are more distantly related to the VirB/D4 proteins [48]. T4SSs are involved in effector translocation, horizontal DNA transfer to other bacteria and eukaryotic cells, in DNA uptake from or release into the extracellular milieu or in the spread of conjugative plasmids [49]. Genome sequence analyses suggest that for *L. pneumophila *T4SSs play an important role for adaptation and virulence as each genome encodes several T4ASSs in addition to the essential T4BSS Dot/Icm discussed above. We identified in each strain either F-type or P-type T4ASSs or both. Figures 2 and Figure 3 show the organization of the structural genes encoding these systems, their organization and their localization (chromosomal or plasmid). The F-type T4ASSs are all predicted to encode a complete T4SS core as well as the essential gene products for pilus assembly and mating pair stabilization that appears to be involved in DNA transfer. They show homology and colinearity with the *tra*-region of the *E. coli *F plasmid [50] and with the recently described *tra *region of *Rickettsia belii *[51]. In *L. pneumophila *strain Philadelphia (Tra5) and *L. longbeachae *strain NSW (Tra6), where the system has a chromosomal localization, it is inserted in a tRNA gene and flanking repeats are present as well as a gene coding for an integrase, suggesting that these T4SSs are mobile (Figure 2). Furthermore, comparison of amino acid identities revealed that the Tra- region on the *L. pneumophila *strain Paris plasmid (Tra1) shows much higher identity with the Tra region located on the *L. longbeachae *plasmid (Tra4) than with those of the different *L. pneumophila *strains (Paris-Tra1, Lens-Tra3 or Lorraine-Tra2) (Figure 2). Thus these systems seem to be transferred horizontally via plasmids but are also able to integrate in the genome similar to what was reported for the Lvh-region [52].

The F-type T4SS encode long, flexible pili that allow donors to mate in liquid and on solid media with equal efficiencies [53]. In contrast P-type T4SS like described in *P. aeuroginosa *encode short and rigid conjugative pili that allow surface mating. Homologues to this system are also present in the *Legionella *genomes. They were initially described in two genomic islands of *L. pneumophila *strain Corby (Figure 3; Trb1 and Trb2) [54]. We show here that they are also present in the chromosomes of *L. pneumophila *strain Lorraine (Trb3) and *L. longbeachae *NSW150 (Trb4) (Figure 3). Again for all T4SS regions flanking repeats are found suggesting mobility, and protein identity values and GC-content values of the *tra*-*trb *genes are higher than the genomic average (38%), supporting again horizontal and not vertical transmission.

Another intriguing feature of these regions is that several transposases and phage related proteins are present in each of the *tra *clusters as well as genes coding for homologues of a putative phage repressor protein (PrpA) and for homologues of LvrA, LvrB and LvrC, first described for the Lvh region of *L. pneumophila*. LvrC is a homologue of CsrA, a protein crucial for the regulation of the switch between replicative and transmissive phase of *L. pneumophila *[55]. It is tempting to assume that these CsrA homologues are implicated in the regulation of the mobility of these islands. Possibly, dependent on the growth phase and/or on metabolic cues *L. pneumophila *might excise these islands as multiple copies could be advantageous in certain conditions, or perhaps allow high frequencies of DNA transfer leading to fast and efficient adaptation to new conditions. The genomic features of these islands suggest a particular mechanism of mobility, which will be interesting to investigate.

#### b) The *L. pneumophila *genomes encode systems specific for protection against invading DNA and stabilization of large genomic fragments

Bacteria have developed multiple methods of protection against mobile genetic elements or bacteriophages. An example for acquired phage specific immunity is clustered regularly interspaced short palindromic repeats (CRISPR) loci [56]. Another type of protection may be conferred by toxin-antitoxin (TA) systems. Bacterial TA systems are small genetic modules composed of a toxin and antitoxin. While toxins are always proteins, antitoxins are either RNAs (type I and III) or proteins (type II) [57]. These systems were first described for being dedicated to plasmid maintenance. Several lines of research indicate that chromosomal TA systems might serve as protection against mobile genetic elements such as plasmids and phages. However, recent studies have shown that type II systems are also involved in the stabilization of large genomic fragments and of integrative conjugative elements [57]. Interestingly, type II TA systems are thought itself to be part of the mobilome and to move from one genome to another through horizontal gene transfer [57].

Genome analyses identified several TA and CRISPR systems. Interestingly, we identified only type II TA systems of which all except two are in a chromosomal location (Table 6). However, of the 18 chromosomal encoded TA systems identified at least 14 are located on putative genomic islands or mobile genetic elements. The two most frequently found TA systems in the *L. pneumophila *genomes are homologues of the HigAB and RelEB systems. HigAB was first described in the *Vibrio cholerae *superintegron where it encodes mRNA cleaving enzymes and can stabilize plasmids [58]. RelEB was shown, when introduced into the *E. coli *chromosome to prevent deletion of flanking DNA and thus to diminish large scale genome reduction [59]. The same function was shown for the ParED system of *Vibrio vulinificus*, homologues of which are also present in one of the *L. pneumophila *genomes (Table 6). Thus, the different *L. pneumophila *TA systems might be important for stabilization of plasmids and integrative conjugative elements and for protection against invasion of plasmids, phages, or other mobile genetic elements.

**Table 6:** Genes encoding putative toxin-antitoxin systems in six *L. pneumophila *genomes

*L. pneumophila *strains						
**Toxin-antitoxin **	**Paris**	**Lens**	**Philadelphia**	**Corby**	**Lorraine**	**HL06041035**

***higA***		*lpl2833 *(96)*	*lpg2914 *(96)			*lpv3285 *(96)
***higB***		*lpl2834 *(87)*	*lpg2915 *(103)			*lpv3286 *(103)
						
***higA***		*lpl1092 *(93)*				
***higB***		*lpl1093 *(107)*				
						
***higA***	*lpp0064 *(434)*				*lpo0072 *(432)*	
***higB***	*lpp0065 *(79)*				*lpo0073 *(79)*	
						
				*lpc2112 *(312)		
		*lpl2291 *(102)*	*lpg2369 (102)*	*lpc2113 *(37)		*lpv2676 *(102)*
**Similar to *hipA***	*lpp2427 *(78)*	*lpl2292 *(312)*	*lpg2370 *(312)	*lpc2114 *(65)	*lpo2551 *(115)*	*lpv2677 *(310)*
						
***yhvA***					*lpo1074 *(168)*	
***sohA***					*lpo1075 *(115)*	
						
***relE***	*plpp0090 *(83)	*lpl1587 *(82)*				
***relB***	*plpp0089 *(95)	*lpl1588 *(85)*				
						
***relE***		*lpl1084 *(84)*		*lpc2177 *(93)*	*lpo0120 *(93)*	
***relB***				*lpc2178 *(88)*	*lpo0119 *(86)*	
						
***parE***						*lpe2361 *(98)*
***parD***						*lpe2360 *(84)*
						
***pemK***					*lpo0114 *(106)	

*TA systems located on putative genomic islands; In parenthesis length of the corresponding protein

The CRISPR/cas system was shown to provide resistance against invading viruses and plasmids and has been identified in many bacteria and archea [60]. CRISPR/cas loci are also present in the *L. pneumophila *genomes of strains Paris, Lens, Alcoy and 130 b but are absent from strains HL06041035 and Lorraine. According to the cas genes, the CRISPR locus of Paris is closely related to that of strain 130 b. In contrast the one of strain Lens located on the plasmid is closely related to the chromosomal CRISPR locus of strain Alcoy as previously described [61]. Strain Lens carries a second CRISR locus on the chromosome; however, it does not seem to be functional like the one encoded by strain Alcoy. Probably strong protection against invading phages is not extremely important, as not all *L. pneumophila *strains contain CRISPR loci. This may be related to their intracellular life style or that despite their widespread occurrence in aquatic environments only few bacteriophages that specifically infect *Legionella *seem to exist [62].

#### c) Accessory genome of strains Lorraine and HL 0604 1035

In order to get insight in the genetic basis of the two newly sequenced strains, possibly implicated in their different disease frequencies (Lorraine is an newly emerging endemic clone and strain HL 0604 1035 is a *L. pneumophila *Sg1 strain never isolated from disease) we analyzed the specific gene content of each of these strains more in depth. Strain HL 0604 1035 contains 92 and strain Lorraine 148 genes without homology to any gene of the other five *L. pneumophila *strains sequenced of which the majority (60 in strain HL 0604 1035 and 73 in strain Lorraine) code for proteins of unknown function (Additional file 2, Tables S2 and additional file 3, Table S3). Among the genes in these two genomes that lack an ortholog in the other sequenced *L. pneumophila *genomes, about 50% are clustered on three large genomic islands. One genomic Island (GI-HL1) of 45 kb spans from *lpv2637 *to *lpv2691*. It is bordered by a Met tRNA gene and encodes a phage related integrase. A second putative mobile element (GI-HL2) of 27 kbs contains the region from *lpv0193 *to *lpv0226*. It is bordered at one side by an integrase and a reverse transcriptase (*lpv0225*) and on the other side by a prophage Rac integrase and a phage excisionase. Strain Lorraine contains also a large genomic island (GI-Lo1) of 69 kb that spans from *lpo2442 *to *lpo2531*. It is inserted in a Met tRNA gene, contains a phage related integrase and flanking repeats of 72 nts. Additional, smaller genomic islands seem to be present, however, their borders are difficult to define. Thus most of the strain specific genes seem to be acquired by HGT through mobility of genomic islands.

Only for few of the specific genes a putative function can be predicted like genes coding for proteins involved in sugar and nucleotide metabolism, for uridine diphosphoglucuronate 5'-epimerase or for an UDP-glucose 6-dehydrogenase. Furthermore a specific ANK motif containing protein and a leucine reach repeat protein are present in strain HL 0604 1035. In strain Lorraine we identified mainly specific metabolic enzymes like a putative flavanone 3-dioxygenase, an enzyme involved in flavonoids metabolism and in biosynthesis of phenylpropanoids, which are secondary metabolites of plants and algae. In addition, *lpo2614 *is predicted to encode a kynurenine-oxoglutarate transaminase, an enzyme that is part of the tryptophan metabolism and *lpo2960 *codes for a putative glycolate oxidase that catalyses the conversion of glycolate and oxygen to glyoxylate and hydrogen proxide. *lpo2502 *codes a homologue of CsbD, a general stress response protein of *Bacillus subtilis *[63]. However, the best BLASTp hit is with the *Protochlamydia amoebophila *homologue, an *Acanthamoeba *sp. symbiont [64]. Probably this gene has been acquired by HGT between these two bacteria within their amoeba host. Quite surprisingly, we identified a gene coding a putative methyl-accepting chemotaxis sensory transducer (*lpv1770*) although all *L. pneumophila *strains analyzed to date do not encode chemotaxis systems. This gene shares 71.34% amino acid identity with Llo3301 of *L. longbeachae *a protein that is part of its chemotaxis system [28] also present in *L. drancourtii *[65]. Probably a common ancestor encoded a chemotaxis system that was lost in *L. pneumophila *through a deletion and degradation process.

#### d) Shared genome of the epidemic strains Paris and Lorraine

A search for genes shared by the two endemic strains but absent in all other strains identified only three genes that fulfilled these criteria and for which a function could be predicted. These encode the alpha, beta and gamma subunits of a putative thiocyanate hydrolase (*lpo1236, lpo1237*, *lpo1238 and lpp1219, lpp1220, lpp1221*). Most interestingly, these strains are both common in France and strain Paris is also world-wide distributed [10] suggesting a better niche adaptation. Indeed, thiocyanate compounds are used for cleaning water circuits and these strains are thus probably able to better resist these treatments [66]. Furthermore, strain Alcoy that is responsible for several outbreaks and many cases of Legionnaires' disease in Spain, also contains these genes [61]. The genes coding the putative thiocyanate hydrolase have a GC content of 41-43%, which is significantly higher than the average G+C content of the *L. pneumophila *genome, which is 38%. When searching for the closest homologues according to BLAST searches we identified them in the genomes of *Rhodococcus opacus *strain B4 and *Nocardia farcinica *spp. These two are high G+C Gram-positive bacteria belonging to the *Actinomycetales*, which are phylogenetically not closely related to *Legionella *suggesting that *L. pneumophila *acquired these genes by horizontal gene transfer.

Taken together, the analysis of the accessory gene content showed again that *L. pneumophila *genomes show high plasticity due to mobile genetic elements and HGT. No specific virulence related genes explaining their different disease frequencies have been identified. However, the identification of a specific thiocyanate hydrolase might explain the wide distribution of strains Paris and Lorraine as it may allow them to better adapted to artificial water systems.

### Evolutionary genomics

#### Phylogenetic reconstruction reveals extensive recombination

To analyze the relationship among the six different *L. pneumophila *strains a phylogenetic reconstruction was done based on a multilocus sequence (MLSA) approach using 31 genes selected according to Zeigler [67] (Table 7 and Additional file 4, Table S4). These 31 genes were chosen as they had been shown to be powerful for predicting the relatedness of bacterial genomes [67]. The phylogeny obtained from their concatenated alignment showed a well-resolved topology with bootstrap values over 50%. To ascertain the reliability of the obtained phylogenetic tree we established individual phylogenies for each of the 31 genes. Surprisingly, the incongruence among several gene trees was high. In addition the Consense program results did not support any node to at least 50%. To further investigate these results we undertook a second analysis using a Shimodaira-Hasegawa test and compared the topologies of the individual alignments of each gene and the concatenated alignment of the 31 genes. As shown in Additional file 5, Table S5 the likelihood-based SH test for alternative tree topologies identified striking discordances. A possible explanation for the identified incongruences among the phylogenies obtained in our study is the presence of recombination events.

**Table 7:** Characteristics of the 31 genes used for phylogenetic reconstruction

Gene Name	Product	Label^a^	Function	Length (nts)^a^
*uvrB*	Excinuclease ABC, subunit B	*lpp0086*	DNA replication, recombination, and repair	1992
*pgk*	Phosphoglycerate kinase	*lpp0152*	Glycolysis/gluconeogenesis	1191
*rpoA*	RNA polymerase, alpha subunit	*lpp0419*	Transcription	993
*ffh*	Signal recognition particle protein, GTPase	*lpp0467*	Transport and binding proteins	1377
*serS*	Seryl tRNA synthetase	*lpp0575*	tRNA aminoacylation	1281
*proS*	Prolyl-tRNA synthase	*lpp0749*	tRNA aminoacylation	1710
*glyA*	Serine hydroxymethyltransferase	*lpp0791*	Glycine/serine hydroxymethyltransferase	1254
*dnaB*	Replicative DNA helicase	*lpp0803*	DNA replication, recombination, and repair	1383
*gpi*	Glucose-6-phosphate isomerase	*lpp0825*	Glycolysis/gluconeogenesis	1500
*lig*	DNA ligase	*lpp1020*	DNA replication, recombination, and repair	2022
*cysS*	Cysteinyl-tRNA synthetase	*lpp1271*	tRNA aminoacylation	1371
*trpS*	Tryptophanyl tRNA synthetase	*lpp1399*	tRNA aminoacylation	1215
*aspS*	Aspartyl-tRNA synthetase	*lpp1434*	tRNA aminoacylation	1782
*ruvB*	Holliday junction DNA helicase	*lpp1534*	tRNA aminoacylation	1011
*nrdA*	Ribonucleoside-diphosphate reductase, alpha subunit	*lpp1738*	Deoxyribonucleotide/ribonucleoside metabolism	2829
*recA*	Bacterial DNA recombination protein	*lpp1765*	DNA replication, recombination, and repair	1047
*tig*	Trigger factor	*lpp1830*	Protein folding and stabilization	1332
*lepA*	GTP-binding membrane protein	*lpp1837*	Translation	1833
*metK*	S-adenosylmethionine synthetase	*lpp2004*	tRNA aminoacylation	1149
*dnaJ*	Heat shock protein	*lpp2006*	Protein folding and stabilization	1140
*argS*	Arginyl tRNA synthetase	*lpp2013*	tRNA aminoacylation	1770
*eno*	Enolase	*lpp2020*	Glycolysis/gluconeogenesis	1269
*ftsZ*	Cell division protein	*lpp2662*	Cell division	1197
*uvrC*	Excinuclease ABC, subunit C	*lpp2698*	DNA replication, recombination, and repair	1857
*dnaX*	DNA polymerase III, subunits gamma and tau	*lpp2802*	DNA replication, recombination, and repair	1671
*recN*	DNA repair protein	*lpp2877*	DNA replication, recombination, and repair	1668
*metG*	Methionyl tRNA synthetase	*lpp2941*	tRNA aminoacylation	2013
*rho*	Transcription terminator factor	*lpp3002*	Translation	1262
*atpD*	ATP synthase F1, subunit beta	*lpp3053*	ATP-proton motive force interconversion	1377
*atpA*	ATP synthase, subunit alpha	*lpp3055*	ATP-proton motive force interconversion	1554
*thdF*	GTP binding protein, thiophene oxidation	*lpp3073*	tRNA and rRNA base modification	1341

^a ^with respect to strain Paris, nts nucleotides

**Figure 4:**
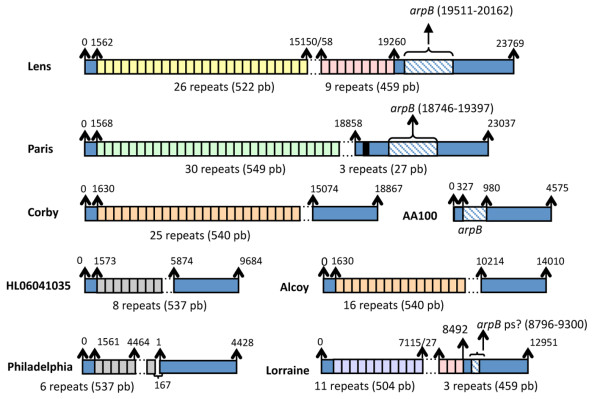
**Schematic representation of the repeat regions present in the *rtxA *gene of *L. pneumophila***. Colored squares represent repeated sequences where the same color corresponds to the same type of repeat. Discontinues lines indicate that the exact number of repeats has not been defined.

With the aim to explore whether recombination events are present in the selected genes we undertook an in depth analysis using the program RDP [68]. Indeed, the analysis of individual genes identified intragenic recombination in 9 of the 31 genes (Table 8). Numerous additional recombination events were detected with the concatenated alignment of the 22 genes for which no intragenic recombination had been shown (Table 8). To minimize false positive recombination events only those that were supported by at least two of the six methods used in RDP were taken into account. However, except one, all were supported by at least three methods. No artifacts resulting of positive selection should be included in this analysis since all of the genes are either informational or operational (housekeeping). Most interestingly, four of the genes in which intragenic recombination was detected are housekeeping genes (*pgk, atpD, ffh, metK*). Housekeeping genes allow to estimate the extent of recombination within bacterial species since presence of recombination in such "normally recombination free genes" is indicative of a high rate of recombination [22]. Similarly antigen-coding genes of *Legionella *were reported to show recombination events [18,69] and certain other genomic regions [17,19,70-72]. Another example of intragenic recombination in *L. pneumophila *is the *rtxA *gene that contains a long tandem repeated domain of variable copy number and sequence [4,10,73]. *rtxA *of strain Lorraine and Corby share the same repeats, whereas the other strains have unique types of repeats. However, when including the newly sequenced strains Lorraine and HL 0604 1035 we found that repeats of the same type are shared by HL 0604 1035 and Philadelphia and by Lorraine and Lens (Figure 4 and Additional file 6, Table S6), further substantiating high intragenic recombination among strains.

**Table 8:** Intragenic and intergenic recombination in six *L. pneumophila *genomes predicted on individual genes and on combined data using six different methods

			Detection Method					
			
Data set	Event Number	Putative recombinant sequences	RDP	GENECONV	Boot scan	Max chi	Chimaera	SiSscan
*metG*	1	Lorraine, Lens	NS	NS	NS	Yes	Yes	Yes
*dnaX*	1	Philadelphia	NS	NS	Yes	Yes	Yes	Yes
	2	Lens, Lorraine	NS	NS	NS	Yes	Yes	Yes
*proS*	1	HL06041035	Yes	Yes	Yes	Yes	Yes	Yes
	2	Philadelphia	NS	Yes	NS	Yes	Yes	Yes
*cysS*	1	Philadelphia	NS	NS	NS	Yes	Yes	NS
*lig*	1	Lorraine	NS	Yes	Yes	NS	NS	NS
*uvrC*	1	Lens,Philadelphia, Lorraine	NS	NS	NS	Yes	Yes	Yes
*flh*	1	Lens	NS	NS	Yes	Yes	Yes	Yes
	2	Paris, HL06041035	NS	NS	NS	Yes	Yes	Yes
*pgk*	1	Lens	NS	NS	NS	Yes	Yes	Yes
*atpD*	1	Corby	NS	NS	NS	Yes	Yes	Yes

Concatenated	1	Philadelphia	Yes	Yes	Yes	Yes	Yes	Yes
	2	Philadelphia	Yes	Yes	Yes	Yes	Yes	Yes
	3	HL06041035	Yes	Yes	Yes	Yes	Yes	Yes
	4	HL06041035	Yes	Yes	Yes	Yes	Yes	Yes
	5	Philadelphia, Corby, Lorraine	Yes	Yes	Yes	Yes	Yes	Yes
	6	Lens	Yes	Yes	Yes	Yes	Yes	Yes
	7	Paris, HL06041035	Yes	NS	NS	Yes	Yes	NS
	8	Paris	Yes	Yes	Yes	Yes	Yes	Yes
	9	Lens	Yes	Yes	NS	Yes	Yes	Yes
	10	Lens	Yes	Yes	Yes	Yes	Yes	NS
	11	HL06041035	Yes	Yes	NS	Yes	NS	NS
	12	Paris, HL06041035	Yes	Yes	Yes	Yes	Yes	Yes
	13	HL06041035, Lens	NS	Yes	NS	Yes	Yes	Yes
	14	Lens, Lorraine	Yes	NS	NS	Yes	Yes	NS
	15	Paris, HL06041035	Yes	Yes	NS	Yes	Yes	NS
	16	Corby	Yes	NS	NS	Yes	Yes	NS
	17	Lens	NS	Yes	NS	Yes	Yes	NS
	18	HL06041035, Paris	Yes	NS	NS	Yes	Yes	Yes
	19	Corby	Yes	NS	NS	Yes	Yes	NS
	20	Lorraine	Yes	Yes	NS	NS	NS	Yes
	21	Lens	Yes	NS	Yes	NS	NS	Yes
	22	Corby	Yes	NS	Yes	Yes	NS	Yes
	23	Lens	NS	Yes	NS	Yes	NS	NS
	24	Lens	NS	Yes	NS	Yes	NS	Yes
	25	Philadelphia	Yes	NS	NS	Yes	Yes	Yes

NS = non significant result. Yes = significant result with p-value ≤0.05 (where P is the highest acceptable probability value of recombination occurrence).

**Figure 5:**
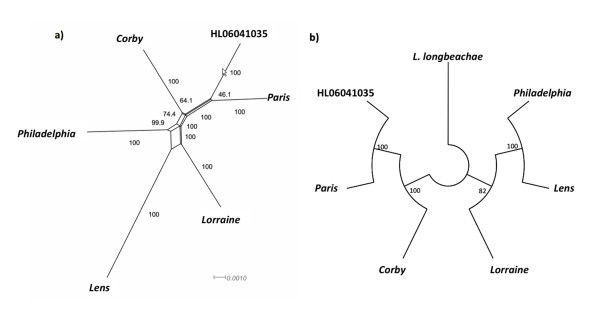
**Phylogenetic relationships of the 6 *L. pneumophila *strains analyzed**. a) Neighbor-net constructed from a concatenation of 31 genes from 6 *L. pneumophila *strains under a GTR model, with associated bootstrap values. b) Likelihood tree topology of *L. pneumophila *strains and the outgroup *L. longbeachae *based on orthologous genes present in all strains/species concatenated.

To reconstruct the phylogenetic history of the species *L. pneumophila *we used thus the concatenated alignment of the 31 genes described above. It gave a topology with high bootstrap support, however recombination bias may result in high support for the wrong tree. To avoid possible bias we thus analyzed the concatenated alignment of the 31 genes using a split tree decomposition that allows a more realistic representation of the phylogenetic relationships. Furthermore we constructed a classical bifurcating tree using the highest possible number of genes [all orthologs among the six strains with (1867 genes) and without (2434 genes) *L. longbeachae *as outgroup]. As shown in Figure 5 the Splits Decomposition phylogeny is network-like suggesting incompatible partitions within sequence data, which commonly arise from recombination. Although the phylogeny based on the orthologous genes can also be affected by recombination, the high number of informative sites included in this data set, should allow recovering the correct history of the species as it has been shown previously for other closely related bacterial species [74].

Taken together, in contrast to previous studies, which reported that the species *L. pneumophila *is a clonal population [13,14] our results show clearly that a high recombination rate shapes the *L. pneumophila *genomes. This finding is in line with the natural competence of *L. pneumophila*. However, some worldwide distributed *L. pneumophila *clones have been described (*e.g*. [10]), suggesting that *L. pneumophila *is able to develop a unique genetic population structure within a particular region or environment as reported recently [72].

#### Recombination of large chromosomal regions of over 200 kbs among *L. pneumophila *strains

**Figure 6:**
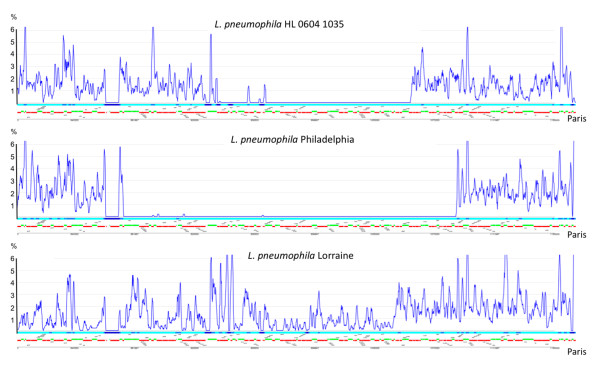
**Distribution of single-nucleotide polymorphisms (SNPs) along 330 kb of the genomes of *L. pneumophila *HL 0604 1035, Philadelphia and Lorraine**. The number of SNPs (y axis) is plotted according to the position of the corresponding 500 bp fragment on the strain Paris chromosome (x axis). A straight blue line indicates 0 polymorphism between the two strains. Numbers on the scale bar indicate the percentage of polymorphism. The green (+ strand) and red (- strand) lines depict the corresponding genes.

Our recombination analysis revealed not only intragenic recombination events but also intergenic recombination as recombination was detected when using the entire alignment even with only recombination free genes (Table 8). This finding may be explained by the recombination of fragments encompassing several genes or multiple recombination events involving smaller tracts along the genome. To test this hypothesis we used a method recently developed for the analysis of *Streptococcus agalactiae *genomes [75]. In order to identify patterns of recombination, nucleotide substitutions between strains were counted in sliding windows across the previously defined core chromosome representing 15 possible pair wise comparisons. Each pair wise comparison revealed highly conserved regions (<0.05% polymorphism on average) and less-conserved regions (>0.7% polymorphism), suggesting the occurrence of recombinational exchanges. When analyzing the different strains in depth we identified in each genome several regions with very low polymorphisms (below 0.05%) suggesting that DNA exchange of these fragments has occurred between the different *L. pneumophila *strains. Most interestingly, the two French strains Paris and HL 0604 1035 that are present since several years in France show 15 regions of a size between 10 and 99 kbs that have very low polymorphism and thus seem to have been exchanged between them (Additional file 7, Figure S1). In contrast when comparing strain Lens with the other 5 genomes analyzed here, very few regions with low polymorphism, two with strain HL 0604 1035 and one with strain Lorraine, were detected. Furthermore, no DNA exchanges seem to have occurred with strains Corby, Philadelphia or Paris. This indicates that strains that are frequent in the same environment (*e.g*. strain Paris and HL 0604 1035) show high rates of DNA exchange probably by conjugation as suggested for *Streptococcus agalactiae *[75] and *Enterococcus fecalis *[76]. In contrast strain Lens, which has been identified to date only twice, in Lens (France) and in Germany, very few DNA transfers with the studied *L. pneumophila *strains seem to have taken place. Furthermore, some regions may be transferred also between several strains. Figure 6 shows the distribution of single-nucleotide polymorphisms (SNPs) along 330 kb of the genome of *L. pneumophila *HL 0604 1035, Philadelphia and Lorraine as compared to the same region in the genome of strain Paris. We identified a region of 213 kbs a SNP frequency of 0.005%. Except an indel of 158 bs that shows higher polymorphism, only 11 SNPs are present in this region. This fragment may have evolved by conjugative transfer and recombination between strains Philadelphia and Paris. Among others, this region carries the genes necessary for lipopolysaccaride biosynthesis, that are also part of the smaller fragment that has been exchanged with strain HL 0604 1035. Our analyses suggest, that in addition to frequent intragenic recombination also recombination and horizontal transfer of large chromosomal fragments is taking place and shapes the chromosomes of *L. pneumophila*.

## Conclusion

Analysis of the genome sequences of six *L. pneumophila *strains shows that the genomes of this environmental pathogen evolve by frequent HGT and high recombination rates. Most interestingly, these events take place between eukaryotes and prokaryotes and among different strains and species of *Legionella*. A genome-wide map analysis of nucleotide polymorphisms among these six strains demonstrated that each chromosome is a mosaic of large chromosomal fragments from different origins suggesting that exchanges of large DNA regions of over 200 kb have contributed to the genome dynamics in the natural population. The many T4SS might be implicated in exchange of these fragments by conjugal transfer. Plasmids also play a role in genome diversification and are exchanged among strains and circulate even between different species of *Legionella*. Importantly, plasmids seem to excise and integrate into the genome probably depending on environmental cues. However, *L. pneumophila *encodes also several toxin anti-toxin that might help to stabilize certain mobile genetic elements. In the near future, the analyses of 100 s of genomes thanks to new generation sequencing combined with molecular studies should provide further clues about the genetic mechanisms and the evolutionary forces that shape the *Legionella *genomes.

## Methods

### Bacterial strains and sequence accession numbers

The strains sequenced in this study are *L. pneumophila *strain Lorraine [EMBL: FQ958210, EMBL:FQ958212] and *L. pneumophila *HL 0604 1035 [EMBL:FQ958211]. Strain Lorraine was isolated in 2004 from a patient and was recently described as a newly emerging endemic clone [26]. *L. pneumophila *strain HL 0604 1035 (ST 734, Bellingham subgroup of the Dresden panel) was isolated in 2006 from a water supply system in a French hospital that it is colonizing since more than 10 years.

### Sequencing and assembly

The complete genome sequence of *L. pneumophila subsp. pneumophila *strain HL06041035 (A) and strain Lorraine (B) were determined using a Sanger/pyrosequencing hybrid approach. A shotgun library was constructed with 10kb size fragments, obtained after mechanical shearing of the total genomic DNA, and cloned into vector pCNS (pSU derived). Sequencing with vector-based primers was carried out using the ABI 3730 Applera Sequencer. A total of 20736 (A) and 21888 (B) reads (~4 fold-coverage) were analyzed and assembled with 502731 (A) and 555541 (B) reads (~15 fold-coverage) obtained with Genome Sequencer GS20 (Roche Applied Science). For the assembly, we used the Arachne "HybridAssemble" version (Broad Institute, http://www.broad.mit.edu) that combines the contigs obtained with 454 sequencing with Sanger reads. To validate the assembly, the Mekano interface (Genoscope), based on visualization of clone links inside and between contigs, was used to check the clone coverage and misassemblies. In addition, the consensus was confirmed using Consed functionalities http://www.phrap.org: the consensus quality and the high quality discrepancies. The finishing step was achieved by PCR, primer walking and *in vitro *transposition technology (Template Generation System™ II Kit; Finnzyme, Espoo, Finland), and a total of 930 (A) and 999 (B) sequences (109, 165 and 656 respectively for *L. pneumophila subsp. pneumophila *strain HL06041035 and 62, 204 and 733 respectively for *L pneumophila subsp. pneumophila *str. Lorraine) were needed for gap closure and quality assessment.

### Sequence analysis and annotation

The two newly sequenced *L. pneumophila *genomes were integrated into the MicroScope platform [77] to perform automatic and expert annotation of the genes, and comparative analysis with the other *L. pneumophila *strains already published. In addition the annotations of the previously published genomes were updated. The system integrates, for each predicted gene, the results of multiple bioinformatics methods (Blast result on UniProt and specialized genomic data, InterPro, COG, PRIAM, synteny group computation using the complete bacterial genomes available at NCBI RefSeq, etc; more information on the syntaxic and functional annotation process is given in [78]). In addition, many genomic and metabolic comparative tools are also available [77]. For details see https://www.genoscope.cns.fr/agc/microscope/home/index.php.

### Definition of orthologous genes

To define orthologous chromosomal genes among the different *L. pneumophila *strains, pseudogenes and mobile elements were not taken into account due to the difficulty of ortholog assignment for these genes. Putative orthologous relations were defined as gene couples fulfilling two criteria: (i) having a bidirectional best hit (BBH) with an alignment threshold of 55% identity over at least 60% of the query sequence and target size (ii) and being in synteny. Subsequently, putative genes without any orthologous relation due to reduced identity percentage were integrated in a pre-existing orthologue group if they were flanked by orthologous genes showing gene order conservation (microsynteny). A final step of manual curation was carried out for each doubtful case.

### Sequence alignments

For each gene of the selected data set, the nucleotide sequence was aligned based on the amino acid sequence using *tranalign/*EMBOSS package http://emboss.sourceforge.net/. Subsequently genes were concatenated in different data sets.

### Identification of eukaryotic like proteins and eukaryotic domain carrying proteins

Eukaryotic domains were identified by analyzing the results obtained for all genes using the Interpro database that is integrated in MAGE. For the identification of eukaryotic like proteins we developed a new method. First we constructed two databases, one containing all and only eukaryotic sequences retrieved from public databases and a second one containing all and only prokaryotic sequences. From the second database we excluded the proteins of bacterial genera for which eukaryotic like protein-domains have been found in high proportions (e.g. parasites of protozoa) or bacterial genera that are reported to establish a symbiotic relationship with amoeba (for a detailed list see Additional file 8, Table S7). Those proteins, that showed a better, normalized blast score against eukaryotic proteins than to those present in the prokaryotic database were retrieved as eukaryotic like proteins. Parameters established for blast were: minimum identity: 25%; minimum ratio avec query: 60%; minimum ratio avec target: 50%. The final results were manually checked.

### Phylogenetic Analysis

For phylogentic reconstruction of the *L. pneumophila *strains analyzed in this work several data sets were used: (i) 31 housekeeping genes described to be essential for all prokaryotes were selected based on the study of Zeigler [67] (Table 7 and Additional file 9, Figure S2) for a multi locus sequence analysis (MLSA) approach for which gene each was analyzed individually and as a concatenated alignment, (ii) a concatenated alignment of 2434 orthologous genes present in all analyzed *L. pneumophila *strains (iii) a concatenated alignment of 1867 orthologous genes present in all analyzed *L. pneumophila *strains and in the selected out group, *Legionella longbeachae *strain NSW150. An analysis of genetic divergence was performed using DNAsp vs 5.00.07 [79] using the 31 selected housekeeping genes. For phylogenetic reconstruction maximum likelihood (ML) methods were used to infer phylogenetic relationships for all data sets. Prior to ML analyses, a DNA substitution model for each gene or data set was selected using Modeltest v3.06 [80] and the Akaike information criterion. ML heuristic searches were performed using 500 random taxon-addition replicates with tree bisection and reconnection (TBR) and branch swapping. ML bootstrap support was determined using 1000 bootstrap replicates. The ML best trees were rooted on *L. longbeachae *when added. A network reconstruction was done for the same data set (i) using SplitsTree4 (version 4.10) [81]. The NeighborNet method and the GTR distance model were used to create the network.

### Congruence test

The 31 genes selected for a MLST approach were tested for the significance of topological differences in the obtained phylogenetic trees using several methods. The first approach was based on the consensus of individual gene trees. The consensus tree was inferred using the CONSENSE program in the PHYLIP package http://evolution.genetics.washington.edu/phylip.html applying the extended majority rule. Secondly we tested the significance of topological differences in phylogenetic trees using the Shimodaira-Hasegawa (SH) test. The SH test compares the likelihood score (-lnL) of a given data set across its ML tree *versus *the -lnL of that data set across alternative topologies, which in this case are the ML phylogenies for other data sets. The differences in the -lnL values are evaluated for statistical significance using 1000 replicates based on resampling estimated with the log-likelihood (RELL) method (PAUP version 4.0b10; http://paup.csit.fsu.edu/. We applied the test using all the trees obtained with individual genes, with the concatenated alignment against the alignment of each individual gene and with the alignment of all the 31 genes concatenated.

### Recombination analysis

The 31 genes selected for a MLST approach and its corresponding concatenated alignment, were screened for the presence of putative recombination events by using RDP 2.0b08 [82]. This program identifies recombinant sequences and recombination breakpoints applying several methods. We selected six of them; two phylogenetic methods (which infer recombination when different parts of the genome result in discordant topologies): RDP [68], 2000) and Bootscanning [83]; and four nucleotide substitution methods (which examine the sequences either for a significant clustering of substitutions or for a fit to an expected statistical distribution): Maxchi and Chimaera [84], GeneConv [85] and Sis-scan [86]. We considered only those recombination events in our analysis that were identified by at least two methods. The common settings for all methods were (i) to consider sequences as circular, (ii) a statistical significance of P < 0.05, and (iii) a Bonferroni correction for multiple comparisons implemented in RDP.

## Abbreviations

ANK: ankyrin motif; CRISPR: Clustered regularly interspaced short palindromic repeats; HGT: horizontal gene transfer; ML: maximum likelihood; nt: nucleotide; Sg1: serogroup 1; T4SS: Type IV secretion system; T2SS: Type II secretion system;

## Competing interests

The authors declare that they have no competing interests.

## Authors' contributions

LGV and CB designed the study. SJ and JE supplied material and expertise; VB and BV performed genome sequencing; LGV and CR performed the genome annotation and analysis work, CM and RZ set up the LegioScope database. LGV and CB drafted and wrote the manuscript. All authors contributed to and approved the final manuscript.

## Supplementary Material

Additional file 1 **Table S1: Nucleotide identity of 140 selected Dot/Icm substrates of strain Philadelphia and of their orthologs in the *L. pneumophila *strains analyzed in this study**. Click here for file

Additional file 2 **Table S2: Genes specific of strain HL 0604 1035 with respect to strains Paris, Lens, Philadelphia, Corby and Lorraine**. Click here for file

Additional file 3 **Table S3: Genes specific of strain Lorraine with respect to strains Paris, Lens, Philadelphia, Corby and HL0604 1035**. Click here for file

Additional file 4 **Table S4: Summary of genetic diversity parameters for the 31 selected *L. pneumophila *genes used to establish the phylogeny**. Click here for file

Additional file 5 **Table S5: Results for the SH Test of alternative topologies for the 6 analyzed *L. pneumophila *strains**. Click here for file

Additional file 6 **Table S6: Conserved domains and repeats of the *rtxA *gene in 8 *L. pneumophila *strains**. Click here for file

Additional file 7 **Figure S1 - Distribution of single-nucleotide polymorphisms (SNPs) along the genome of *L. pneumophila *HL 0604 1035 as compared to strains Lens, Philadelphia, Corby and Lorraine**. The number of SNPs (y axis) is plotted according to the position of the corresponding 500 bp fragment on the strain Paris chromosome (x axis). A straight blue line indicates 0 polymorphism between the two strains. Numbers on the scale bar indicate the percentage of polymorphism. Yellow blocks indicate chromosomal regions with a SNP number lower than 0,005%. Click here for file

Additional file 8 **Tables S7 - List of bacterial genera removed from our prokaryotic database**. Click here for file

Additional file 9 **Figure S2: Distribution of the 31 genes selected for establishing the phylogeny of *L. pneumophila *species**. The coordinates are given with respect to the chromosome of *L. pneumophila *strain Paris. Numbers next to gene names indicate the first position of the corresponding gene starting from the origin of replication. Click here for file
