# Supplementary material for: Extensive recombination events and horizontal gene transfer shaped the Legionella pneumophila genomes
Source: BMC Genomics. 2011 Nov 1;12:536. doi: 10.1186/1471-2164-12-536 (PMC3218107; doi:10.1186/1471-2164-12-536)
Supplement: Additional file 1 — Table S1: Nucleotide identity of 140 selected Dot/Icm substrates of strain Philadelphia and of their orthologs in the L. pneumophila strains analyzed in this study. [file 1471-2164-12-536-S1.DOC]

**Table S1.** Nucleotide identity of 140 selected Dot/Icm substrates of strain Philadelphia and their orthologs in the *L. pneumophila* strains analyzed in this study
