# Supplementary material for: Extensive recombination events and horizontal gene transfer shaped the Legionella pneumophila genomes
Source: BMC Genomics. 2011 Nov 1;12:536. doi: 10.1186/1471-2164-12-536 (PMC3218107; doi:10.1186/1471-2164-12-536)
Supplement: Additional file 2 — Table S2: Genes specific of strain HL 0604 1035 with respect to strains Paris, Lens, Philadelphia, Corby and Lorraine. [file 1471-2164-12-536-S2.DOC]

**Table S2**: Genes specific of strain HL 0604 1035 with respect to strains Paris, Lens, Philadelphia, Corby and Lorraine.

* at least 30% of aminoacid identity over a minimum lenght of 0.8% of the smallest protein
