# Supplementary material for: Extensive recombination events and horizontal gene transfer shaped the Legionella pneumophila genomes
Source: BMC Genomics. 2011 Nov 1;12:536. doi: 10.1186/1471-2164-12-536 (PMC3218107; doi:10.1186/1471-2164-12-536)
Supplement: Additional file 4 — Table S4: Summary of genetic diversity parameters for the 31 selected L. pneumophila genes used to establish the phylogeny. [file 1471-2164-12-536-S4.DOC]

**Table S4:** Summary of genetic diversity parameters for the 31 selected *L. pneumophila* genes used to establish the phylogeny.

* Range is based on the size of the genes in the different *L. pneumophila* strains
