# Supplementary material for: Extensive recombination events and horizontal gene transfer shaped the Legionella pneumophila genomes
Source: BMC Genomics. 2011 Nov 1;12:536. doi: 10.1186/1471-2164-12-536 (PMC3218107; doi:10.1186/1471-2164-12-536)
Supplement: Additional file 5 — Table S5: Results for the SH Test of alternative topologies for the 6 analyzed L. pneumophila strains. [file 1471-2164-12-536-S5.DOC]

**Table S5 :** Results for the SH Test of alternative topologies for the 6 analyzed *L.* *pneumophila* strains.

Values denote p-values of a given data set across its own ML tree as well as across each of the alternative trees (the gene trees and the concatenated tree) *P<0.05
