# Supplementary material for: Extensive recombination events and horizontal gene transfer shaped the Legionella pneumophila genomes
Source: BMC Genomics. 2011 Nov 1;12:536. doi: 10.1186/1471-2164-12-536 (PMC3218107; doi:10.1186/1471-2164-12-536)
Supplement: Additional file 6 — Table S6: Conserved domains and repeats of the rtxA gene in 8 L. pneumophila strains. [file 1471-2164-12-536-S6.DOC]

**Table S6:** Conserved domains and repeats of the *rtxA* gene in 8 *L. pneumophila* strains
