# Supplementary figures and images for: Extensive recombination events and horizontal gene transfer shaped the Legionella pneumophila genomes
Source: BMC Genomics. 2011 Nov 1;12:536. doi: 10.1186/1471-2164-12-536 (PMC3218107; doi:10.1186/1471-2164-12-536)

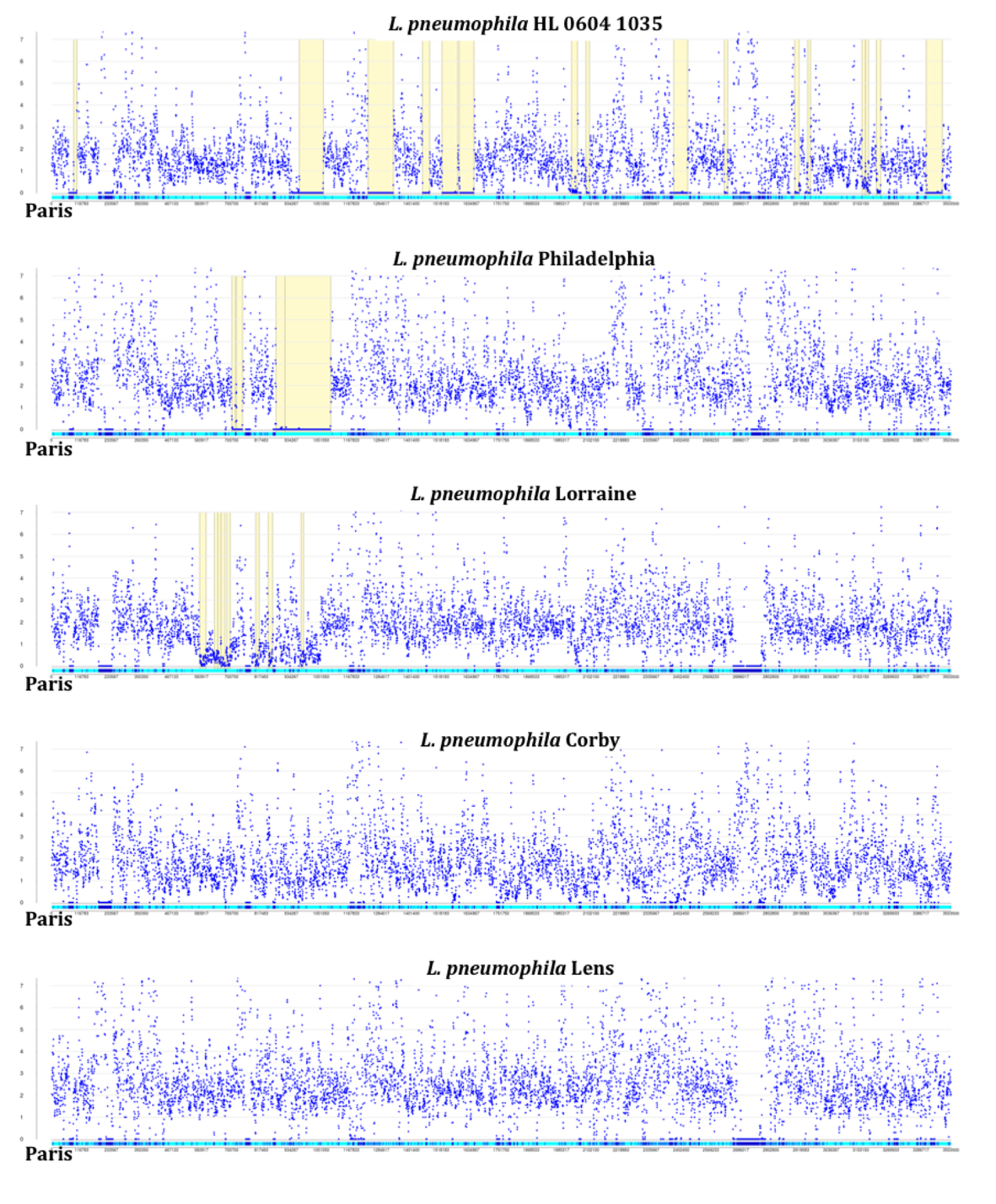

Supplement: Additional file 7 — Figure S1 - Distribution of single-nucleotide polymorphisms (SNPs) along the genome of L. pneumophila HL 0604 1035 as compared to strains Lens, Philadelphia, Corby and Lorraine. The number of SNPs (y axis) is plotted according to the position of the corresponding 500 bp fragment on the strain Paris chromosome (x axis). A straight blue line indicates 0 polymorphism between the two strains. Numbers on the scale bar indicate the percentage of polymorphism. Yellow blocks indicate chromosomal regions with a SNP number lower than 0,005%. [file 1471-2164-12-536-S7.PNG]

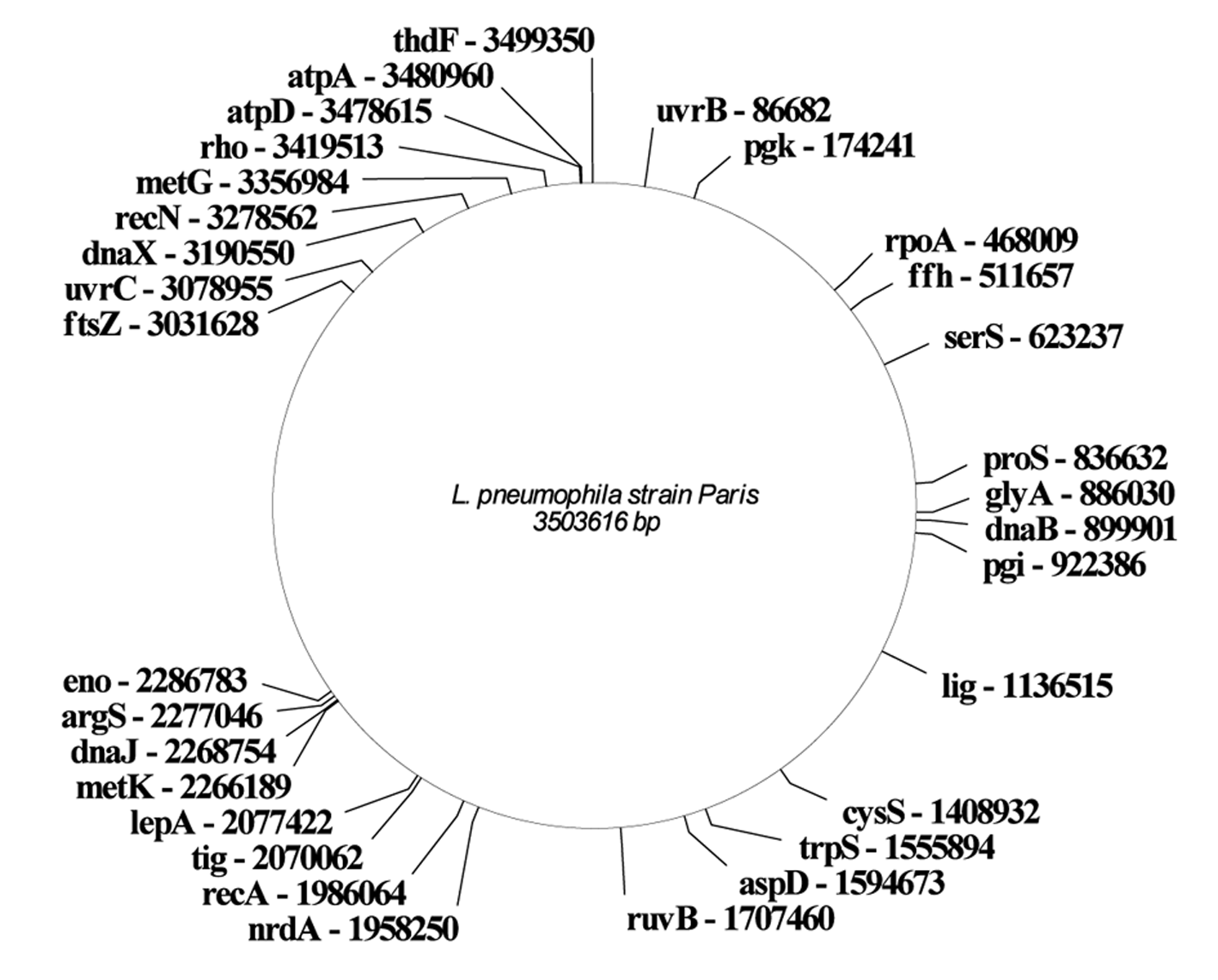

Supplement: Additional file 9 — Figure S2: Distribution of the 31 genes selected for establishing the phylogeny of L. pneumophila species. The coordinates are given with respect to the chromosome of L. pneumophila strain Paris. Numbers next to gene names indicate the first position of the corresponding gene starting from the origin of replication. [file 1471-2164-12-536-S9.PNG]
