# Supplementary material for: Extensive recombination events and horizontal gene transfer shaped the Legionella pneumophila genomes
Source: BMC Genomics. 2011 Nov 1;12:536. doi: 10.1186/1471-2164-12-536 (PMC3218107; doi:10.1186/1471-2164-12-536)
Supplement: Additional file 8 — Tables S7 - List of bacterial genera removed from our prokaryotic database. [file 1471-2164-12-536-S8.DOC]

**Table S7:** List of bacterial genera removed from our prokaryotic database

*Acinetobacter, Actinobacterium, Aeromonas, Afipia, Agrobacterium, Amoebophilus, Anaplasma , Bacillus, Bosea, Bradyrhizobium, Brevundimonas, Brucella, Burkholderia, Caedibacter, Chlamydia , Chlamydophila , Comamonas, Coxiella, Criblamydia, Delftia, Ehrlichia, Enterobacter, Escherichia, Flavobacterium, Flexibacter, Francisella, Helicobacter, Klebsiella, Legionella , Listeria, Mezorhizobium, Molibuncus, Mycobacterium , Neochlamydia, Odyssella, Orientia, Paracaedibacter, Parachlamydia, Pasteurella, Porphyromonas, Procabacter, Protochlamydia, Pseudomonas, Ralstonia, Rhodococcus, Rickettsia, Rothia, Salmonella, Serratia, Simkania, Sphingobacterium, Sphingomonas, Staphylococcous, Stenotrophomonas, Streptococcus, Vibrio, Wolbachia, Wolinella*

References used for creating this list:

1) References for bacterial genera with genomes enriched in eukaryotic domains or eukaryotic like proteins:

1. Cazalet C, Rusniok C, Bruggemann H, Zidane N, Magnier A, Ma L, Tichit M, Jarraud S, Bouchier C, Vandenesch F *et al*: **Evidence in the *Legionella pneumophila* genome for exploitation of host cell functions and high genome plasticity**. *Nat Genet* 2004, **36**(11):1165-1173.

2. Cole ST, Brosch R, Parkhill J, Garnier T, Churcher C, Harris D, Gordon SV, Eiglmeier K, Gas S, Barry CE, 3rd *et al*: **Deciphering the biology of *Mycobacterium tuberculosis* from the complete genome sequence**. *Nature* 1998, **393**(6685):537-544.

3. Ogata H, La Scola B, Audic S, Renesto P, Blanc G, Robert C, Fournier PE, Claverie JM, Raoult D: **Genome sequence of *Rickettsia bellii* illuminates the role of amoebae in gene exchanges between intracellular pathogens**. *PLoS Genet* 2006, **2**(5):e:76.

4. Schrammeijer B, Risseeuw E, Pansegrau W, Regensburg-Tuink TJ, Crosby WL, Hooykaas PJ: **Interaction of the virulence protein VirF of *Agrobacterium tumefaciens* with plant homologs of the yeast Skp1 protein**. *Curr Biol* 2001, **11**(4):258-262.

5. Seshadri R, Paulsen IT, Eisen JA, Read TD, Nelson KE, Nelson WC, Ward NL, Tettelin H, Davidsen TM, Beanan MJ *et al*: **Complete genome sequence of the Q-fever pathogen *Coxiella burnetii***. *Proc Natl Acad Sci U S A* 2003, **100**(9):5455-5460. Epub 2003 Apr 5418.

6. Wu M, Sun LV, Vamathevan J, Riegler M, Deboy R, Brownlie JC, McGraw EA, Martin W, Esser C, Ahmadinejad N *et al*: **Phylogenomics of the Reproductive Parasite *Wolbachia pipientis* wMel: A Streamlined Genome Overrun by Mobile Genetic Elements**. *PLoS Biol* 2004, **2**(3):E69.

7. Duchaud E, Boussaha M, Loux V, Bernardet JF, Michel C, Kerouault B, Mondot S, Nicolas P, Bossy R, Caron C *et al*: **Complete genome sequence of the fish pathogen *Flavobacterium psychrophilum***. *Nat Biotechnol* 2007, **25**(7):763-769.

8. Klasson L, Walker T, Sebaihia M, Sanders MJ, Quail MA, Lord A, Sanders S, Earl J, O'Neill SL, Thomson N *et al*: **Genome evolution of *Wolbachia* strain wPip from the *Culex pipiens* group**. *Mol Biol Evol* 2008, **25**(9):1877-1887.

9. Cho NH, Kim HR, Lee JH, Kim SY, Kim J, Cha S, Darby AC, Fuxelius HH, Yin J, Kim JH *et al*: **The *Orientia tsutsugamushi* genome reveals massive proliferation of conjugative type IV secretion system and host-cell interaction genes**. *Proc Natl Acad Sci U S A* 2007, **104**(19):7981-7986.

10. Horn M, Wagner M: **Bacterial endosymbionts of free-living amoebae**. *J Eukaryot Microbiol* 2004, **51**(5):509-514.

11. Beare PA, Unsworth N, Andoh M, Voth DE, Omsland A, Gilk SD, Williams KP, Sobral BW, Kupko JJ, 3rd, Porcella SF *et al*: **Comparative genomics reveal extensive transposon-mediated genomic plasticity and diversity among potential effector proteins within the genus *Coxiella***. *Infect Immun* 2009, **77**(2):642-656.

12. Hayashi T, Makino K, Ohnishi M, Kurokawa K, Ishii K, Yokoyama K, Han CG, Ohtsubo E, Nakayama K, Murata T *et al*: **Complete genome sequence of enterohemorrhagic *Escherichia coli O157:H7* and genomic comparison with a laboratory strain K-12**. *DNA Res* 2001, **8**(1):11-22.

13. Schmitz-Esser S, Tischler P, Arnold R, Montanaro J, Wagner M, Rattei T, Horn M: **The genome of the amoeba symbiont "Candidatus *Amoebophilus asiaticus*" reveals common mechanisms for host cell interaction among amoeba-associated bacteria**. *J Bacteriol* 2010, **192**(4):1045-1057.

14. Ogura M, Perez JC, Mittl PR, Lee HK, Dailide G, Tan S, Ito Y, Secka O, Dailidiene D, Putty K *et al*: ***Helicobacter pylori* evolution: lineage- specific adaptations in homologs of eukaryotic Sel1-like genes**. *PLoS Comput Biol* 2007, **3**(8):e151.

15. Rikihisa Y, Lin M: ***Anaplasma phagocytophilum* and *Ehrlichia chaffeensis* type IV secretion and Ank proteins**. *Curr Opin Microbiol* 2010, **13**(1):59-66.

2) References for bacterial genera resistant to amoeba-infection :

10. Horn M, Wagner M: **Bacterial endosymbionts of free-living amoebae**. *J Eukaryot Microbiol* 2004, **51**(5):509-514.

16. Schmitz-Esser S, Toenshoff ER, Haider S, Heinz E, Hoenninger VM, Wagner M, Horn M: **Diversity of bacterial endosymbionts of environmental *acanthamoeba* isolates**. *Appl Environ Microbiol* 2008, **74**(18):5822-5831.

17. Evstigneeva A, Raoult D, Karpachevskiy L, La Scola B: **Amoeba co-culture of soil specimens recovered 33 different bacteria, including four new species and *Streptococcus pneumoniae***. *Microbiology* 2009, **155**(Pt 2):657-664.
